# A Multi-Method Approach for Quantification of Surface Coatings on Commercial Zinc Oxide Nanomaterials

**DOI:** 10.3390/nano10040678

**Published:** 2020-04-03

**Authors:** Filip Kunc, Oltion Kodra, Andreas Brinkmann, Gregory P. Lopinski, Linda J. Johnston

**Affiliations:** National Research Council Canada, Ottawa, ON K1A 0R6, Canada; Filip.Kunc@nrc-cnrc.gc.ca (F.K.); Oltion.Kodra@nrc-cnrc.gc.ca (O.K.); Andreas.Brinkmann@nrc-cnrc.gc.ca (A.B.)

**Keywords:** nanoparticles, surface functional group quantification, thermogravimetric analysis, quantitative NMR, X-ray photoelectron spectroscopy

## Abstract

Surface functionalization is a key factor for determining the performance of nanomaterials in a range of applications and their fate when released to the environment. Nevertheless, it is still relatively rare that surface groups or coatings are quantified using methods that have been carefully optimized and validated with a multi-method approach. We have quantified the surface groups on a set of commercial ZnO nanoparticles modified with three different reagents ((3-aminopropyl)-triethoxysilane, caprylsilane and stearic acid). This study used thermogravimetric analysis (TGA) with Fourier transform infrared spectroscopy (FT-IR) of evolved gases and quantitative solution ^1^H nuclear magnetic resonance (NMR) for quantification purposes with ^13^C-solid state NMR and X-ray photoelectron spectroscopy to confirm assignments. Unmodified materials from the same suppliers were examined to assess possible impurities and corrections. The results demonstrate that there are significant mass losses from the unmodified samples which are attributed to surface carbonates or residual materials from the synthetic procedure used. The surface modified materials show a characteristic loss of functional group between 300–600 °C as confirmed by analysis of FT-IR spectra and comparison to NMR data obtained after quantitative release/extraction of the functional group from the surface. The agreement between NMR and TGA estimates for surface loading is reasonably good for cases where the functional group accounts for a relatively large fraction of the sample mass (e.g., large groups or high loading). In other cases TGA does not have sufficient sensitivity for quantitative analysis, particularly when contaminants contribute to the TGA mass loss. X-ray photoelectron spectroscopy and solid state NMR for selected samples provide support for the assignment of both the functional groups and some impurities. The level of surface group loading varies significantly with supplier and even for different batches or sizes of nanoparticles from the same supplier. These results highlight the importance of developing reliable methods to detect and quantify surface functional groups and the importance of a multi-method approach.

## 1. Introduction

Most applications of nanomaterials require that the surface of the as-produced material be chemically modified [1]. Some surface modifications are introduced directly during the synthesis, for example to add capping agents and control the particle size. Others are added in a second step aimed at imparting colloidal stability and minimizing aggregation, tuning optical properties, ensuring biocompatibility and biotargeting, or facilitating incorporation in polymer matrices. The nanomaterial surface chemistry is a key factor in determining the behavior of the material in applications and its environmental and human health impact [2,3]. Nevertheless, it is rare that the surface groups are quantified, particularly for commercial nanomaterials, and in some cases the chemical nature of the surface and the presence of possible contaminants are not addressed [4]. It should be noted that this is not due to a lack of methods for surface chemistry analysis [5,6,7,8,9,10,11,12,13,14,15,16]. However, the choice of appropriate method and its range of applicability and sensitivity are frequently not clear. An additional complication is provided by the presence of surface accessible and inaccessible ligands for some nanomaterials [17]. Distinguishing between these ligands is particularly important in cases where the modified nanomaterial is used for further functionalization, as for example to add biotargeting ligands [18]. 

Recent work from our group has focused on a detailed comparison of methods for identifying and quantifying surface functional groups on a variety of commercial and in-house synthesized silica nanoparticles (NPs) with varying size, surface functionality and preparation method [17,19,20]. The methods include a silica dissolution/quantitative solution NMR (qNMR) approach for measuring total functional group content, colorimetric assays using ninhydrin and nitrobenzaldehyde probes to assess surface accessible groups, solid state NMR (ssNMR) of silicas in which the functional group is conjugated to a fluorinated probe, X-ray photoelectron spectroscopy (XPS) and thermogravimetric analysis (TGA). These studies have focused on optimizing the various methods and understanding their sensitivity, ease of use and, most importantly, limitations. This work has indicated that for silicas which can be readily dissolved releasing covalently attached groups, qNMR is the method of choice based on the widespread availability of solution NMR and the ease of determining both the structure and quantity of the functional group. However, qNMR measures total functional group content and there is value in comparing the data to colorimetric assays that rely on binding to or reaction with an optical reporter. Such assays can also be readily accessed in most laboratories and will measure only functional groups that are accessible to the probe used, of particular relevance for assessing the efficiency of reactions that introduce further surface functionality. Thermogravimetric analysis is an easy to use method, and when combined with analysis of FT-IR spectra of evolved gases, provides some structural information. Nevertheless, it suffers from relatively low sensitivity and for silica is complicated by release of water over a broad temperature range and in some cases loss of contaminants introduced during synthesis. Both ssNMR and XPS provide useful corroborating information, particularly in cases where the surface chemistry is more complex or contaminants are present, although neither can be considered routine, generally accessible methods. 

In the present study we have used qNMR and TGA with FT-IR of evolved gas for analysis of 20 different samples of commercially sourced zinc oxides (ZnO) from four suppliers and two materials from the European Union Joint Research Center (EU JRC) Nanomaterial Repository. The physical chemical properties of the latter materials have been examined in detail [21] and they have been widely used for nanotoxicology studies in the Organisation for Economic Co-operation and Development (OECD) testing program and large EU projects [22]. The NPs vary in size and have been surface modified with (3-aminopropyl)triethoxysilane (APTES), caprylsilane or stearic acid. The choice of ZnO was based on its widespread production and use for a diverse range of applications including catalysts, sensors, coatings, sunscreens and cosmetics, which makes it an important material for consideration of regulatory guidelines [23]. Many applications require surface modifications, as for example the use of octylsilane for sunscreens and stearic acid coatings for vulcanisation of rubber. We are particularly interested in testing the applicability of methods developed for release or extraction of common ligands from silica NPs to functionalized metal oxides. The study also aims to address potential TGA issues related to overlapping signals from functional groups and solvent, impurities or contaminants in the sample, which is a significant limitation for silica. As in previous work we have found it useful to compare results to XPS and ssNMR for selected samples in order to resolve some of the complexities encountered with both TGA and qNMR. The conclusions of the present work illustrate an approach to quantifying nanoparticle surface chemistry that will be beneficial for development of applications and nanotoxicology studies. 

## 2. Materials and Methods 

### 2.1. Materials

Bare and functionalized ZnO nanopowders were acquired from four different manufacturers: US Research Nanomaterials (‘USRN’, Houston, TX, USA); Skyspring Nanomaterials (‘Sky’, Houston, TX, USA); BASF (‘BASF’, Edison, NJ, USA); and Nano and Amorphous Materials (‘NAM’, Katy, TX, USA). All samples were received as dry powders in plastic containers or bags and were transferred to glass containers and stored under ambient conditions. Samples are named according to the manufacturer/supplier (e.g., NAM, USRN, Sky, BASF) and each sample is numbered sequentially (e.g., NAM-01, NAM-02) in order of mention in the text. Modified samples have an additional designation to indicate the surface chemistry/coating (e.g., NH_2_, C8 and C18 for propyl amine, capryl and stearic acid groups). Note that we have no way of identifying the bare sample that was used for surface modification for most of these commercial nanomaterials, so each sample was assigned a different number. In one case the manufacturer confirmed that the bare sample (NAM-01) was used as a precursor for functionalization (NAM-03-NH_2_). Two samples were provided courtesy of JRC: samples JRC-01 and JRC-02-C8 are NM-110 and NM-111 in the JRC characterization report and were later renamed by the JRC to JRCNM62101a and JRCNM1101a [21]. 

Deuterium oxide (99.9%), deuterium chloride (33% in D_2_O), sodium deuteroxide (10 M in D_2_O), methanol-d_4_ (99.9%), DMSO-d_6_ (99.9%), pyridine-d_4_ (99.9%), trifluoroacetic acid-d (TFA-d) 99.9%, TraceCERT maleic acid (99.94% maleic acid mass fraction), and TraceCERT potassium phthalate monobasic (99.92% mass fraction) were purchased from Sigma-Aldrich (Oakville, ON, Canada). 

### 2.2. In-House Stearic Acid Modification 

Bare ZnO sample BASF-01 (1 g) was dispersed in ~ 100 mL ethanol) by sonication in an ultrasonic bath for 15 min. Stearic acid (0.1 mmol, 28.4 mg) was dissolved in 1 mL of ethanol and the solution was combined with the prepared dispersion. This was further sonicated for 5 minutes. The ethanol was then evaporated in a rotavap and the powder obtained (NRC-01-C18) was dried under vacuum for 24 h. A control sample was prepared by the same procedure omitting the addition of stearic acid.

### 2.3. Quantitative NMR Sample Preparation

#### 2.3.1. ZnO-APTES Modification

Acidic dissolution method: The powdered sample was weighed (6–12 mg) into an empty microcentrifuge tube (Eppendorf 1.5 mL safe-lock, Fisher Scientific, Ottawa, ON, Canada) with precision ± 0.01 mg. DCl (0.65 mL, 0.4 M in D_2_O) was added, the sample was dispersed in an ultrasonic bath, placed into a heated shaker (Ther-Mix heated mixer, Vitl Life Science Solutions, Ashland, VA, USA) and shaken at 1200 RPM at 45 °C for the described amount of time (3–24 h). The cloudy dispersion turned transparent after a few min indicating that the ZnO NP disintegration is rapid. The sample was then cooled to room temperature and an exact mass of D_2_O solution of potassium hydrogen phthalate (internal standard) was added prior to the qNMR measurement which was conducted within 48 h.

Basic extraction method: The powdered sample was weighed (6–12 mg) into an empty microcentrifuge tube. NaOD (0.7 mL, 0.4 M in D_2_O) was added, the sample was dispersed in an ultrasonic bath, placed into the heated shaker and shaken at 1200 RPM at 45 °C for 24 h. The sample was cooled to room temperature and centrifuged for 8 min at 14.6k RPM. The supernatant (0.65 mL) was collected, and stored in a separate Eppendorf tube prior to the qNMR analysis which was conducted within 48 h. The remaining supernatant was discarded, and the pellet was dispersed in a fresh aliquot of NaOD (0.7 mL, 0.4 M in D_2_O) and the process was repeated 1 or 2 times to test if further aminopropylsilane could be hydrolysed from the ZnO surface. 

#### 2.3.2. ZnO-Caprylsilane Modification

The powdered sample was weighed (6–12 mg) into an empty microcentrifuge tube or a 1 mL glass ampoule with precision ± 0.01 mg. Methanolic NaOD solutions (at concentrations of 0.2, 0.4, and 0.8 M) were prepared by addition of NaOD stock solution (10 M in D_2_O) to methanol-d_4_. Methanolic NaOD (0.65 mL) was combined with the ZnO powder, the sample was dispersed in an ultrasonic bath and placed in the heated shaker and shaken at 1200 RPM at 45 °C for the described amount of time (1–3 days). Performing the experiment in plastic microcentrifuge tubes for more than 48 h should be avoided, since some NMR-detectable alkyl contaminants start to leach out after this time. The samples were then cooled to room temperature and centrifuged at 5000 RPM for 5 min to separate the solid powder from the solution. The supernatant was collected with a Pasteur pipette, and stored in an Eppendorf tube prior to the NMR analysis which was conducted within 48 h. The remaining pellet was redispersed into a fresh portion of methanolic NaOD (0.65 mL) and the process was repeated 1 or 2 times. 

#### 2.3.3. ZnO-Stearic Acid Modification

Dissolution method: The powdered sample was weighed (6–12 mg) into a 1 mL glass ampoule with precision ± 0.01 mg. Then 0.65 mL of methanol-d_4_ and TFA-d (8 or 16%) were added and the sample was mildly shaken. After ~ 5 min, the powder dissolved completely leaving a transparent solution. Since contact of TFA solution with plastic should be avoided, the glass ampoule was capped with aluminium foil. The sample was stored in the glass ampoule prior to qNMR analysis. The sample stability was confirmed at time points of 1 h, 24 h and 2 weeks after the dissolution.

Extraction method: The powdered sample was weighed (6–12 mg) into an empty microcentrifuge tube. Then, 0.65 mL of DMSO-d6, methanol-d_4_ or 16% pyridine-d_4_ in methanol-d_4_ was added to the powder which was dispered in an ultrasonic bath. The sample was placed in the heated shaker and shaken at 1200 RPM at 45 °C for 24 h. The sample was cooled to room temperature and centrifuged at 5000 RPM for 5 min to separate the solid powder from the solution. The supernatant was collected with a Pasteur pipette, and stored in an Eppendorf tube prior to qNMR analysis. The remaining pellet was redispersed in a fresh portion of solvent (0.65 mL) and the process was repeated one or two times. 

### 2.4. Quantitative Solution NMR Experiments

All experiments were performed at 20 °C (± 1 °C) with an Avance III 400 MHz spectrometer (Bruker, Billerica, MA, USA) equipped with a 5 mm BBFO probe. For each analyte sample, the 90° pulse width was calibrated by determining the null signal generated by a 360° pulse width divided by 4 (in μs). The spin-lattice-relaxation times (T_1_) of internal standards (maleic acid or potassium hydrogen phthalate) in specific solvents were sourced from the guidance on TraceCERT^®^ certified reference materials for quantitative NMR provided by the supplier. A relaxation delay of at least 7× T_1_ (standard) was used in the experiment [(T_1_ (standard) > T_1_ (analyte)]. The maximum value for the receiver gain was obtained. The ^1^H-NMR spectrum was then recorded using the 90° pulse program with the following parameters: two dummy transients, 16–32 transients, and 20.0 ppm spectral width with 6.1 ppm transmitter offset. The acquired free induction decays (FIT) were processed by Fourier transformation and phase- and baseline-corrected manually by a fifth-order polynomial fit. Analyte diagnostic signals were identified and integrated and normalised for the number of protons. Integrals that deviated by >20% from the average of other integrals were excluded. The average of the remaining integrals was used to calculate the final content *c* expressed in µmol of functional group per g of material and standard deviation (individual values shown in Tables S1–S10) using the following equation:

$$c\;\left[\frac{\mu mol}{g}\right]=\frac{(I_{b}\times n_{Hb}/\left(I_{a}\times n_{Ha}\right))\times \frac{m{\left(st\right)}^{\prime }\;}{Mw\;}}{m_{s}}\times 10^{6}$$

where $I_{a}$ is the integral value of the internal standard resonance, $I_{b}$ is the integral value of the analyte signal, *n_H_*_a_ is number of hydrogen atoms corresponding to the internal standard signal, *n_Hb_* — number of hydrogen atoms corresponding to the analyte signal, $m{\left(St\right)}^{\prime }$ is mass of internal standard added in to the sample in *g*, $M_{w}$ is molecular weight of internal standard, and $m_{s}$ is the weighed mass of the sample in mg. The final values were calculated as an average of individual experiment replicates with respective standard deviation. 

### 2.5. Solid-State NMR Experiments

All solid-state NMR experiments were performed on a Bruker 200 MHz Avance spectrometer (Billerica, MA, USA) employing a 7 mm Bruker double resonance probehead. About 350 mg of bare or stearic acid modified ZnO was loaded into the sample holders. The probe was double-tuned to ^1^H and ^13^C Larmor frequencies of 200.5 MHz and 50.4 MHz, respectively. During all experiments the sample was rotated at a frequency of 5 kHz around an axis at an angle of 54.74° with respect to the external magnetic field. Both ^13^C direct polarization (DP) and cross-polarization (CP) experiments were performed employing SPINAL-64 decoupling [24] with a ^1^H nutation frequency of 76 kHz during signal acquisition. The ^1^H and ^13^C 90 pulses were given by 3.5 µs and 4.1 µs, respectively. For the CP experiments a contact pulse with a duration of 2 ms was employed with ^1^H and ^13^C nutation frequencies of 52 kHz and 47 kHz, respectively. The values for the ^1^H and ^13^C T1 constant of the CH_2_ groups in the stearic acid modified ZnO were estimated to be 2.7 s and 49 s. respectively. A relaxation delay of at least 7× T_1_(^13^C) was employed for the ^13^C DP experiments. The concentration of stearic acid functional groups was quantified by integrating the CH_2_ region in the ^13^C DP spectra and employing benzoic acid as external standard. 

### 2.6. TGA Experiments

Experiments were conducted using either a Iris TG209 F1 or a Jupiter STA449 F1 instrument (both from NETZSCH, Chicago, IL, USA) coupled with a Tensor 27 FT-IR spectrometer (Bruker, Chicago, IL, USA). Temperature and mass calibrations were done as recommended by the manufacturer. In a typical experiment, 20–40 mg of powdered sample was loaded in an empty aluminum oxide crucible that was pre-treated by annealing in a natural gas flame for approximately 30 s. The mass of sample was adjusted to ensure that a total mass loss of at least 1 mg was obtained. The sample was inserted into the instrument under 50 mL min^−1^ argon atmosphere (argon protective 25 mL min^−1^) and left to stabilize the mass for 1 h; the transfer line to the FT-IR was also purged with the same flow of argon. The thermal cycle 25–950 °C (10 °C min^−1^) was then initiated maintaining the same argon flow. For FT-IR results the residence time in the transfer line is ∼2.5 s. All TGA experiments were run against the correction for an empty aluminum oxide crucible in argon atmosphere. Thermograms were processed by first excluding the mass loss below ∼200 °C due to the presence of moisture. The components in the thermograms were separated into three general regions 200–300 °C, 300–600 °C, and > 600 °C; for each sample the temperature range over which each component was measured was adjusted by no more than ± 15 °C to best match the switch between regions in the TGA curve. All mass loss values are expressed as wt %. 

### 2.7. XPS Experiments

XPS was carried out using an Axis Ultra DLD spectrometer (Kratos Analytical, Manchester, UK) with monochromatized Al Kα X-rays. ZnO powder samples were measured directly with three distinct points measured on each sample. Survey spectra over the entire energy range were first obtained in order to estimate the relative atomic composition of the sample and detect any impurities that may be present. High resolution spectra were subsequently acquired in regions corresponding to the strongest core level transitions for the major elements present on these samples (Zn2p, O 1s and C 1s for all samples and Si 2s, Si 2p and N1s where these were observed in the survey). These high-resolution spectra were used for more accurate quantification of the sample composition and also provided additional information on the chemical state of the elements. All energy scales for high resolution spectra were adjusted by determining the shift needed to ensure the Zn 2p3/2 peak was centered at 1021.8 eV. The C1s and O1s regions for the same sample and measurement point were then shifted by this amount. Data analysis was carried out with the CasaXPS software (Casa Software, Teignmouth, UK). For calculating elemental ratios, the integrated area under each peak was determined after subtracting a Shirley background and corrected using Kratos relative sensitivity factors. Decomposition of the C1s and O1s spectra into various components was carried out using mixed Gaussian-Lorentzian (GL30) lineshapes. 

## 3. Results

### 3.1. TGA Analysis of Unmodified ZnO NPs

**Figure 1:**
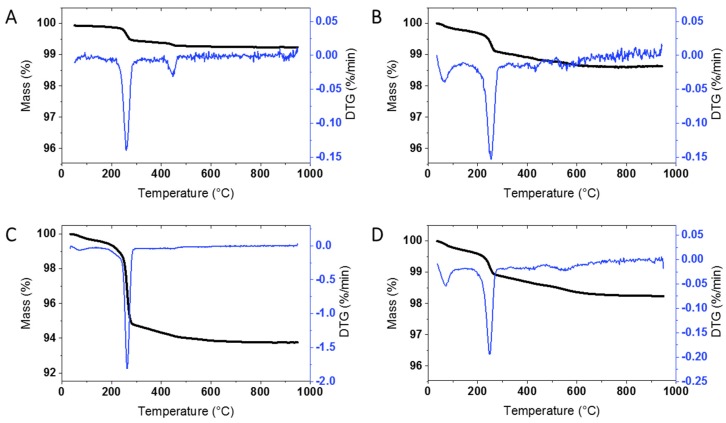
Representative thermograms of bare ZnO samples: (**A**) JRC-01, (**B**) NAM-01, (**C**) USRN-02, and (**D**) USRN-03. Note that the y-axis for (**C**) has a different range for clarity of presentation of the results.

An initial survey of TGA results for at least one unmodified sample from each supplier was carried out in order to determine whether there is significant mass loss that will have to be taken into account for the surface modified samples. Representative results for ZnO from the four suppliers, as well as the JRC, are shown in Figure 1 and Figure S1; the sample codes are provided in the Materials and Methods. JRC-01 shows significant mass loss at ~260 °C (0.5%) and a smaller mass loss at higher temperatures. This can be contrasted to the behavior of BASF-01 sourced from the same supplier as JRC-01 but purchased at the beginning of this study. Here the overall mass loss is considerably lower (Figure 1A vs Figure S1A) without a clearly defined maximum at 260 °C. The original characterization report for JRC-01 reported only a small mass increase for this sample upon heating [21]. However a later study that examined the JRC-01 sample and three batches of unmodified ZnO from the same supplier found that the total mass loss varied from 0.76 (JRC) to 0.93%. An inflection point at ~ 260 °C was also observed and FT-IR spectra showed loss of both carbon dioxide and water; carbon was detected in XPS spectra and was postulated to be from an adventitious carbon source [25]. Overall these results indicate that the organic content of the BASF (JRC) sample may vary with time or sample handling. 

Most of the samples from the three other commercial suppliers (USRN, NAM, Sky) give similar results with a clearly defined mass loss at ~ 260 °C (Figure 1 and Figure S1) and varying levels of mass loss at higher temperatures. Table 1 summarizes the mass loss for each of the bare samples; the thermograms are divided into 3 temperature regions (200–300 °C, 300–600 °C and >600 °C; see Materials and Methods Section) for ease of comparison with the results for the surface modified materials discussed in the following sections. The mass loss from 200–300 °C, with a maximum at 260 °C, varied significantly for the bare samples, from a high of 4.61% for USRN-02 (Figure 1C) to 0.24 for Sky-01 (Figure S1B) and was low with lack of a clear inflection point in USRN-01 and NAM-02. The mass loss between 300–600 °C also varied across the samples, from a low of 0.13% for BASF-01 to a high of 1.6% for USRN-01. The mass loss in this region is expected to overlap with that for most organic coatings or modifications, as outlined below. With the exception of USRN-01, there is minimal mass loss (≤0.21%) above 600 °C. Note that in a few cases there is a small mass loss below 120 °C which is assigned to water but is not included in the Table. Multiple replicates were measured for 4 of the samples with good agreement between runs, with the exception of the high temperature mass loss which had a larger standard deviation for USRN-01 (Table 1). 

**Figure 2:**
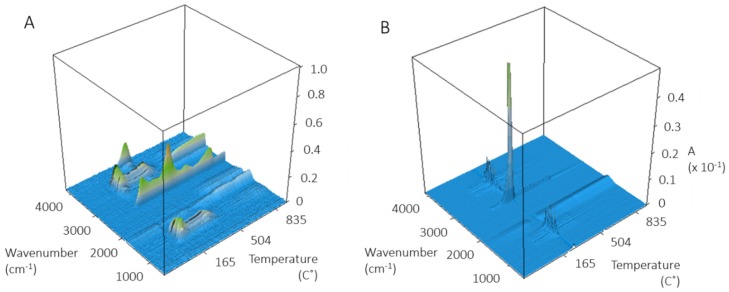
3D FT-IR spectra of evolved gases from the TGA analysis of bare ZnO samples: (**A**) USRN-01, and (**B**) USRN-02. The analysis was carried out in an argon atmosphere. The signal at 3000–3200 cm^-1^ in panel A is due to a background issue.

FT-IR spectra of evolved gases were measured for several ZnO NPs in an attempt to identify the source of the prominent component at 260 °C. The 3D spectra for USRN-01 and USRN-02 (Figure 2) are dominated by loss of CO_2_ at 260 °C with a minor amount of water; only water and CO_2_ are detected across the temperature range examined. USRN-01 which had a larger mass loss between 300–600 °C than the other samples gave more complex 3D FT-IR spectra with loss of both water and CO_2_ across a range of temperatures and the appearance of a peak assigned to ammonia at 965 cm^-1^ at ~340 °C. Ammonia is not detected for USRN-02 or NAM-01 (Figure S5A), suggesting that its presence in USRN-01 at least partly accounts for the large mass loss between 300–600 °C for this sample. Additional FT-IR signals in the 1100–1800 cm^-1^ and 2800–3400 cm^-1^ regions at temperatures above 600 °C for USRN-01 could not be conclusively identified, but are consistent with spectra for polyaromatic hydrocarbons. BASF-01 gave very weak FT-IR signals (not shown) consistent with the very low mass loss for this sample. 

A control experiment using the procedure summarized for extraction of hydrophobic moieties (methanol-d_4_/trifluoroacetic acid; see section below for stearic acid) was carried out for USRN-01 to test for the presence of hydrolysable or extractable material that could be identified by solution NMR. No signals were detected by ^1^H NMR, but there was an additional signal at ~ 160 ppm in the ^13^C-NMR spectrum. Based on the results in Table 1, we hypothesized that the mass loss occurring in the intermediate temperature region (300–600 °C) was most likely to interfere with loss of functional groups from the modified ZnO samples. The loss of material below 300 °C and above 600 °C are likely to be below and above the temperature for functional group loss, based on previous studies. 

### 3.2. qNMR and TGA Analysis of APTES-Functionalized ZnO NPs

**Figure 3:**
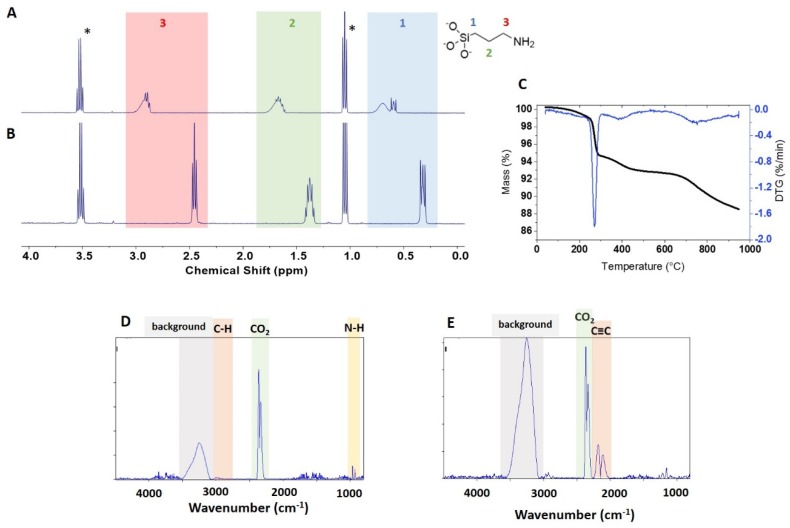
Characterisation of APTES functionalized ZnO sample USRN-05-NH_2_: (**A**, **B**) ^1^H NMR spectra of 3-aminopropylsiloxane moiety after dissolution of the sample in 0.4 M DCl (A) or hydrolysis in 0.4 M NaOD (**B**), (**C**) representative thermogram and (**D**, **E**) FT-IR spectra of evolved gases measured at 345 °C (**D**) and 744 °C (**E**). The signals marked with an asterisk in (A) are due to ethanol. The grey shaded areas (**C**, **D**) are due to background caused by ice condensation in the detector.

The surface loading for APTES-functionalized NPs was first quantified by qNMR. Initial experiments focused on determining appropriate conditions to remove the covalently attached surface groups for qNMR analysis (Tables S1–S3). Our previous studies using NMR for quantification of functional groups on silica were based on the dissolution of the silica matrix in basic D_2_O (NaOD 0.4 M) which released the functional groups for solution NMR analysis and quantification by comparison to an internal standard. Since ZnO is insoluble in basic solution [26] we tested a comparable approach based on dissolving APTES-functionalized zinc oxide in acidic D_2_O (DCl 0.4 M). This did not always lead to the full dissolution of the powder; for example NAM-03-NH_2_ produced a cloudy sample from which solids could be recovered by centrifugation (Table S1). In another case (USRN-05-NH_2_) the NMR spectra showed broad resonances (Figure 3A) which we previously explained as an indication of insufficient dissolution with the 3-aminopropylsiloxane moiety existing in polymerised form [19]. Note that the signals are much weaker for the other APTES-functionalized NPs, making it difficult to assess whether the NMR signals are also broadened. 

To explore the viability of the previous protocol using basic hydrolysis of siloxane bonds, the samples were treated with 0.4 M NaOD for various periods of time at 45 °C (data summarized in Tables S1–S3). The aim was to release 3-aminopropylsiloxane from the NP surface and separate the supernatant from the remaining ZnO solids. This approach provided NMR spectra with well-defined signals (Figure 3B). In order to test whether a single extraction was quantitative some samples were extracted with base a second time or extracted for a longer period of time. For USRN-05-NH_2_ (which has a high APTES loading) this led to recovery of an additional minor amount of 3-aminopropylsiloxane. The quantification of APTES for the various ZnO samples using the basic hydrolysis method is summarized in Table 2. The comparison of data for the acidic hydrolysis and the basic hydrolysis, followed by NMR of the supernatant, are summarized in Tables S1–S3. The amount of recovered functional group is similar for the two methods for the two USRN samples, despite the evidence for incomplete dissolution for the acidic hydrolysis. However, acidic hydrolysis recovers 28% less functional group for NAM-03-NH_2_. Based on the various tests, we conclude that basic hydrolysis and qNMR of the supernatant is the preferred method, although it does require a second hydrolysis step to ensure complete recovery of the functional group, particularly for samples with high amine loading. 

As shown in Table 2, the amine loading is relatively low for two of the APTES-modified samples, with 15 and 35 μmol/g for USRN-04-NH_2_ and NAM-03-NH_2_, respectively. A ten-fold higher loading of 454 μmol/g was obtained for USRN-05-NH_2_. The variability in loading level for samples from a single supplier is similar to our previous results for silica nanoparticles [17,19] and literature results for other nanomaterials [18,25,27]. Note that the more than twenty-fold difference in 3-aminopropyl loading for USRN-05-NH_2_ and USRN-04-NH_2_ cannot be explained by differences in surface area since these samples have nominal diameters of 10–30 and 20 nm, respectively. 

The APTES-functionalized samples were examined by TGA with the expectation that the sample with the highest loading should have a sufficient mass loss for reliable quantification. As shown in Figure 3C, the mass loss for USRN-05-NH_2_ occurs in three distinct regions, corresponding to 200–300 °C, 300–600 °C and >600 °C. The FT-IR spectra of evolved gases showed the presence of CO_2_ and water (Figure S5B), but the C–H bands expected for the loss of functional group were not detected in the expected region (Figure 3D,E), probably due to low abundance. This is consistent with our previous experience with APTES-modified silicas [20]. 

Based on the observation of the 260 °C mass loss for unmodified ZnO samples and the observation of a weak signal consistent with an amine (Figure 3C), we hypothesized that the 300–600 °C mass loss was most likely to be due to to loss of APTES from the surface. The mass loss in this region was estimated to correspond to 381 μmol/g of APTES, in reasonable agreement with the qNMR data (454 μmol/g). Correction of the APTES mass loss using data for NPs of the same size gives a value of 299 μmol/g, considerably lower than the qNMR value and suggesting that the bare sample does not provide a reliable correction. The mass loss at >600 °C is much larger for this sample than the modest changes (~ 0.2%) observed for most of the unmodified ZnO NPs. The FT-IR spectra in this region show typical CO_2_ signals and a minor 2000–2300 cm^-1^ band which occurs in this temperature region in other functionalized samples (Figure 3E). Although it was not possible to identify the probable chemical structure of this component by comparison to the spectral library, a number of molecules with unsaturated C≡C bonds show signals in this region. Note that the large background signal between 3000–3500 cm^-1^ is due to ice condensation in the detector and makes it impossible to detect other signals in this region. 

TGA curves for the remaining APTES-functionalized samples show smaller mass loss in the 300–600 °C range (Figure S2). For USRN-04-NH_2_ the mass loss in this region is 0.9%, the same as that for the mass loss in the equivalent-size bare sample (USRN-02, Table 1). This suggests that that amine loading is low, as observed by NMR (~15 μmol/g); the entire mass loss of 0.9% would correspond to an amine content of ~154 μmol/g). Similarly for NAM-03, the mass loss is 0.36%, similar to the value of 0.41 for a bare sample of the same size from the same supplier. Note that one could possibly improve on the correction if the precursor of the modified sample was available. Both of these samples show only a small mass loss above 600 °C that is similar to that in unmodified samples, suggesting that the mass loss at high temperatures for USRN-04-NH_2_ is somewhat anomalous. 

### 3.3. qNMR and TGA Analysis of Caprylsilane Modified ZnO NPs

Two caprylsilane-functionalized samples were examined using a method similar to the one optimized for APTES-modified ZnO, but with methanol-d_4_ as solvent in order to solubilize the more hydrophobic alkyl group. Tables S4 and S5 summarize the conditions that were investigated to optimize the hydrolysis and release the caprylsilane moiety into the solution. Multiple concentrations of NaOD were tested in order to achieve quantitative hydrolysis while maintaining a sufficiently hydrophobic solvent to solubilize the released caprylsilane. NaOD concentrations of 0.2, 0.4 and 0.8 M were tested with reaction times of 24 and 48 h at 45 °C. A second and third hydrolysis step were also tested in some experiments. Overall the highest recovery of functional group was for samples with 2 hydrolysis steps of 24 h in 0.4 M NaOD, with an additional third hydrolysis step yielding only traces of caprylsilane (Table S4). The recovery obtained for a single 78-h extraction with either 0.4 M or 0.8 M NaOD was ~10% lower, whereas the use of 0.2 M NaOD gave 50–60% of the maximum recovery. This indicates that base concentration rather than time is the more important factor for quantitative release of the caprylsilane. Higher temperatures (85 °C) led to deposition of material on the walls of the glass ampoule and low recovery of the caprylsilane. 

**Figure 4:**
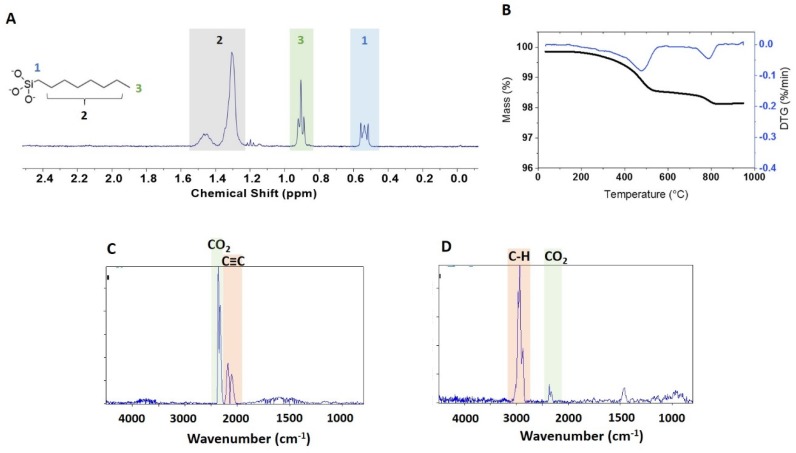
Characterisation of caprylsilane functionalized ZnO sample BASF-02-C8: (**A**) ^1^H NMR spectra of caprylsiloxane moiety dissolved in 0.4 M NaOD in methanol-d_4_, (**B**) representative thermogram and (**C**,**D**) FT-IR spectra of evolved gases measured at 560 °C (**C**) and 807 °C (**D**).

A qNMR spectrum of the extracted functional group is shown in Figure 4A for BASF-02-C8. The JRC-02-C8 and BASF-02-C8 samples (both from supplier BASF) gave the same caprylsilane loading (101, 102 μmol/g), Table 2, suggesting that the surface modification reaction is reproducible for these samples. TGA for these two samples showed mass losses between 300 and 600 °C with an inflection point at ~ 475 °C (Figure 4B and Figrue S3). Making the assumption that this corresponds to loss of the caprylsilane, the mass losses correspond to 98 and 93 umol/g for JRC-02-C8 and BASF-02-C8, after correction for the small mass loss in this region for the corresponding bare samples. The agreement with the qNMR data supports our hypothesis that the mass loss between 300 and 600 °C can be assigned to the caprylsilane. FT-IR spectra for BASF-02-C8 showed C–H signals at 2700–3000 cm^-1^ at temperatures between 300–600 °C (Figure 4C and Figrue S5C), consistent with the loss of alkyl groups, further confirmation that this component is due to loss of the caprylsilane. Neither caprylsilane-modified ZnO sample shows the clear signature for mass loss at 260 °C that was observed for some other samples and there was no detectable loss of CO_2_ at this temperature in the FT-IR spectra (Figure S5C). However, there is additional mass loss at high temperatures that is of variable intensity for the samples examined and that varies from run to run for the same sample. The FT-IR spectra show a large signal due to CO_2_ and the previously mentioned bands at 2000–2300 cm^-1^ (Figure 4D). Note that the original characterization data reported a mass loss of ~ 1% for JRC-02-C8 [21], whereas a later study of the JRC sample and two other batches of the same material reported 2-step mass losses between 2.1 and 2.4% [25]. 

### 3.4. qNMR and TGA Analysis of Stearic Acid Modified ZnO NPs

A number of conditions were tested to remove the surface coating from stearic acid modified ZnO NPs. Initial experiments tested multiple extraction steps (each 24 h at 45 °C) using DMSO and methanol-d_4_ but, ≤10 μmol/g stearic acid was recovered (Table S6). A modified extraction procedure using pyridine-d_4_ in methanol-d_4_ gave a higher recovery of stearic acid from ZnO NPs (43 µmol/g), although this value was still lower than the estimate from TGA (see below) [28]. This may be caused by the proposed strong chemical interaction between ZnO and stearic acid, since some literature reports indicate that stearic acid is covalently bonded to ZnO [29]. Therefore, we investigated a two-phase method in which stearic acid modified ZnO was first dispersed in an organic solvent (toluene, cyclohexane, and chloroform tested) and then combined with HCl (0.4 M) followed by 24 h reflux. After this, both layers appeared transparent, indicating the disintegration of starting material. Due to its high partition coefficient (log P_O/W_ = 8.23), stearic acid should be concentrated in the organic layer. However, after solvent evaporation, NMR analysis revealed the presence of multiple impurities that interfered with the diagnostic signals of stearic acid. These impurities may have been introduced during the reflux or be due to traces of organic solvent and therefore this method was not suitable for quantitative analysis. 

**Figure 5:**
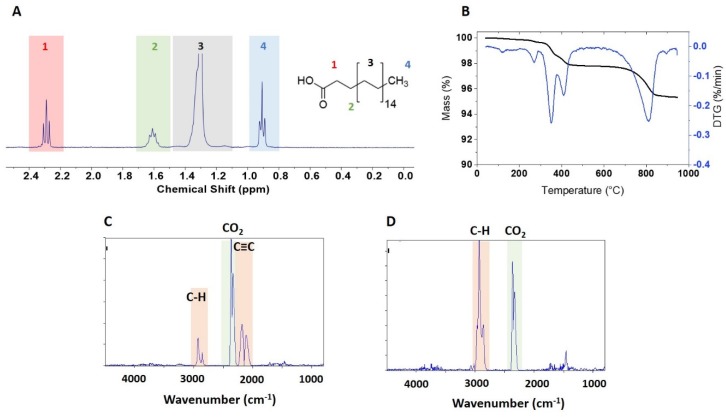
Characterization of stearic acid functionalized ZnO sample NRC-01-C18: (**A**) ^1^H NMR spectra of solubilized stearic acid in 16% TFA-d in methanol-d_4_; (**B**) representative thermogram and (**C**,**D**) FT-IR spectra of evolved gases measured at 407 °C (**C**), and 814 °C (**D**).

An alternate method based on dissolution of the ZnO matrix in mixtures of methanol-d_4_ and trifluoroacetic acid (TFA-d) was tested (Tables S7–S9). This limited the sample manipulation and excluded non-deuterated solvents. The initially cloudy dispersion became transparent which suggests that the core nanomaterial had dissolved. The use of an organic acid is necessary because stearic acid is insoluble in methanol containing even low fractions of water (>5%). Integration of the acquired NMR spectra confirmed the chemical structure of stearic acid and gave values for stearic acid content that were similar to the TGA results (see below). The values obtained by dissolution of three commercial stearic acid-modified ZnO NPs in TFA- methanol-d_4_ are provided in Table 2. This method gave values with relatively high standard deviation for two USRN samples, although the repeatability was reasonably good for Sky-02-C18. In order to further confirm the suitability of the method, a bare ZnO sample (BASF-01) was modified with stearic acid (NRC-01-C18) and analyzed by qNMR (Figure 5A, Table 2, Table S10). This demonstrated recovery of ~90% of the initial stearic acid used for surface modification at several TFA concentrations. Based on this it appears that the TFA concentration used for the experiment is not critical as long as it facilitates dissolution of ZnO. The relatively high standard deviation in the two USRN samples is independent of TFA concentration, and appears to be specifically related to samples from this manufacturer. 

TGA of the four samples modified with stearic acid showed mass loss in 3 regions, 200–300 °C, 300–600 °C and >600 °C, Figure 5B and Figure S4, although with intensities that differed from sample to sample. The low temperature region has a clear maximum at 260 °C for all samples, with a mass loss that varies from ~0.2% (NRC-01-C18) to ~5% (USRN-07-C18, Sky-02-C18); USRN-06-C18 has a small shoulder at ~170 °C that may be due to water or solvent (Figure S4A). As for other samples described above, the FT-IR spectra of NRC-01-C18 and USRN-07-C18 are dominated by loss of CO_2_ and water at low temperatures (Figure S5D,E). Interestingly the unmodified sample BASF-01 does not show a maximum at 260 °C, but there is a clear, although small, mass loss in this region for the stearic acid-modified sample. A control sample prepared by treating BASF-01 with the stearic acid modification conditions in the absence of stearic acid had a mass loss of ~ 0.1% between 200–300 °C. 

The middle temperature region (300–600 °C) has maxima at 355 and 405 °C that are assigned to loss of stearic acid, based on the loss of other organic groups in this region and on the observation of bands consistent with alkyl groups between 2700–3000 cm^-1^ at a temperature of 403 °C in the FT-IR spectra (Figure 5C, Figures S5D,E and S6A). The magnitude of the mass loss above 600 °C is variable for multiple runs of the same sample. Consistent with other functionalized samples, signals at 2000–2300 cm^-1^ start to emerge in the FT-IR spectra for both NRC-01-C18 and USRN-07-C18 above 600 °C (Figure 5D, S6B). These signals are of higher intensity for NRC-01-C18 than for the commercial samples and there is a low intensity alkyl band at 2700–3000 cm^-1^ at temperatures above 600 °C (Figure 5D). However, the continuous release of high boiling point components in the FT-IR may not be related to their desorption from the analysed material; this is more likely to be due to the condensation of these compounds in the TGA-FT-IR transfer line and their slow release at later times during the TGA run. The control sample of BASF-01 that was subjected to the same surface modification conditions in the absence of stearic acid showed only a modest mass loss of 0.34% between 200 and 500 °C.

### 3.5. X-Ray Photoelectron Spectroscopy

XPS data was obtained for selected samples to provide further confirmation of the conclusions from NMR and TGA and to seek additional insight on the source of the mass loss at 260 °C for both bare and modified ZnO and the high temperature mass loss that was significant for several samples. Survey XPS scans for bare ZnO powders (see Table S11) indicated that the main elements present are Zn, O and C, with a small Cl signal (~1–3% atomic composition). Chlorine impurities have previously been reported in XPS of ZnO NPs [25] and are likely due to the procedure used for NP synthesis. While survey scans are useful to rapidly obtain information on the atomic composition of the sample and identify the presence of impurities, high resolution scans of the strongest core levels associated with the major constituents were used for further quantitative analysis as summarized in Table 3. The O/Zn atomic composition ratios are close to 1 (0.99 to 1.06), as expected; a notable exception is USRN-02 which had a significantly higher O/Zn ratio (1.18). All samples showed the presence of a substantial amount of carbon with most of the samples exhibiting C/Zn ratios between 0.39 and 0.67 with no clear correlation between this ratio and the total mass loss measured by TGA for these samples. However, the largest carbon content was for USRN-02 which also had a high O/Zn ratio and a large mass loss at 260 °C, suggesting that this mass loss corresponds to a species containing both C and O. As XPS is a surface sensitive technique and sensitive to adventitious C contamination, as observed previously for silica NPs [17], we assume that the observed C/Zn ratios reflect a mixture of C contamination and carbon-containing impurities in these ZnO samples. 

**Figure 6:**
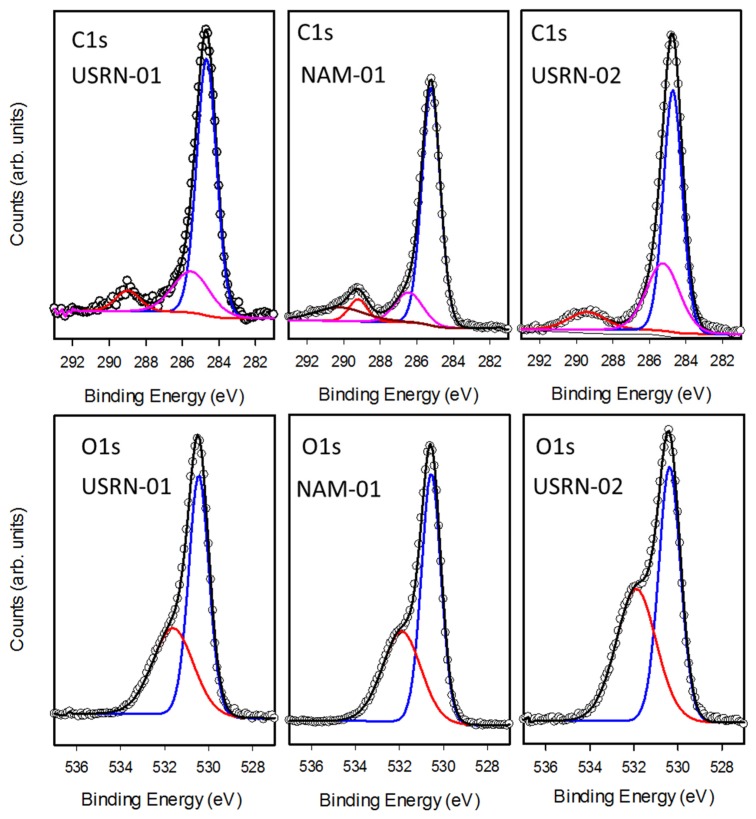
XPS spectra of the C1s (top) and O1s regions for 3 of the unmodified ZnO samples. The spectra are fit with a Shirley background and mixed Gaussian-Lorentzian peaks (GL30) using CASA-XPS software. The variously colored peaks correspond to the components in Table 3; C1s blue (C1), pink (C2), red (C3) and dark red (C4), O1s blue (O2) and red (O3).

The high-resolution scans of the C1s and O1s regions, shown in Figure 6, also reveal additional information on the nature of the chemical species present in these samples. The C1s region is dominated by a peak with maximum intensity at 285 eV along with a smaller peak at 289 eV (Figure 6). Standard curve fitting procedures were used to decompose the spectra into several components, reflecting the different bonding environments for carbon present on the sample. The main peak at 285 eV (C1) is characteristic of carbon-carbon or carbon-hydrogen bonding whereas the shifted peaks (C2 at 286eV, C3 at 289 eV and C4 at 290 eV) are assigned to carbon bound to oxygen. While all the samples exhibited C1, C2 and C3 components, the broad C4 feature was only observed in three of the samples, including NAM-01 in Figure 6. 

The fraction of total carbon assigned to each of the four signals is summarized in Table 3. Based on literature precedent we hypothesize that the C3 and C4 signals are due to carbonate as a range of carbonates have XPS signals at ~ 289–290 eV [30,31]. Carbonate could be introduced by the synthetic procedure used (which is not divulged by the manufacturer) or by adsorption of carbon dioxide [32,33]. An earlier study had provided FT-IR evidence for the formation of surface ZnCO_3_, showing that the amount increased with time [32]. In that study an XPS signal at 286.5 eV was assigned to the carbonate, although this is inconsistent with XPS data for a range of carbonates. In the present work, the BASF-01 sample did exhibit a greater fraction of its carbon signal in the C3 and C4 states in a second measurement performed at a later time, indicating that C–O species may be accumulating on the sample. The O1s region (Figure 6, Table 3) can be fit with two components at 530.5 (O2) and 532 eV (O3) which are assigned to metal oxide and O–C containing moieties (such as carbonates), respectively. Whereas USRN-02 showed an almost equal fraction of O2 (52%) and O3 (48%) signals, for all the other bare particles O2 (58-62%) was significantly higher than O3. This is consistent with USRN-02 exhibiting a greater degree of carbonate (or other C–O containing impurity).

Surface-modified ZnO NPs showed changes that are consistent with introduction of the expected functional groups (Table 4, Table S12). Detection of Si in the silane modified samples was complicated by the presence of Zn plasmon loss features associated with Zn 3p and Zn 3s peaks that are adjacent to the Si 2p and Si2s peaks. Despite this complication Si signals are clearly observed for both APTES and caprylsilane functionalized samples in high resolution spectra (Figure S7). The sharper Si features were separated from the broad Zn plasmon losses for quantification. As seen in Table 4, Si/Zn ratios extracted using the Si 2s and Si 2p peaks are in reasonable agreement. The caprylsilane and APTES samples exhibit similar Si signals, indicative of similar degrees of functionalization. This is consistent with the qNMR and TGA data once the results are converted to surface coverage of functional groups on the particles. For the APTES samples a N1s signal is also observed with the N/Zn ratio comparable to the Si/Zn ratio as expected. O/Zn ratios were higher (1.12–1.45) than those measured for all bare samples, except USRN-02. Similarly, the C/Zn ratios were higher than for bare ZnO samples from the same manufacturer, with the highest values obtained for stearic acid modified samples. The presence of adventitious carbon contamination, as noted for the bare samples above as well as in our previous study of silica particles [17], complicates the use of the C/Zn ratio for quantification of the surface functional groups. However, comparing the C/Zn ratios for the three different stearic acid modified particles shows a trend that mirrors the NMR and TGA results with USRN-06-C18 showing the highest degree of stearic acid loading. The C/Zn ratios were similar for the two caprylsilane modified samples, again consistent with the Si/Zn ratios and the NMR and TGA data, although the O/Zn ratio varied. For APTES, the C/Zn increased by a smaller amount and repeat measurements showed higher variability, presumably due to the lower carbon content in the functional group. 

**Figure 7:**
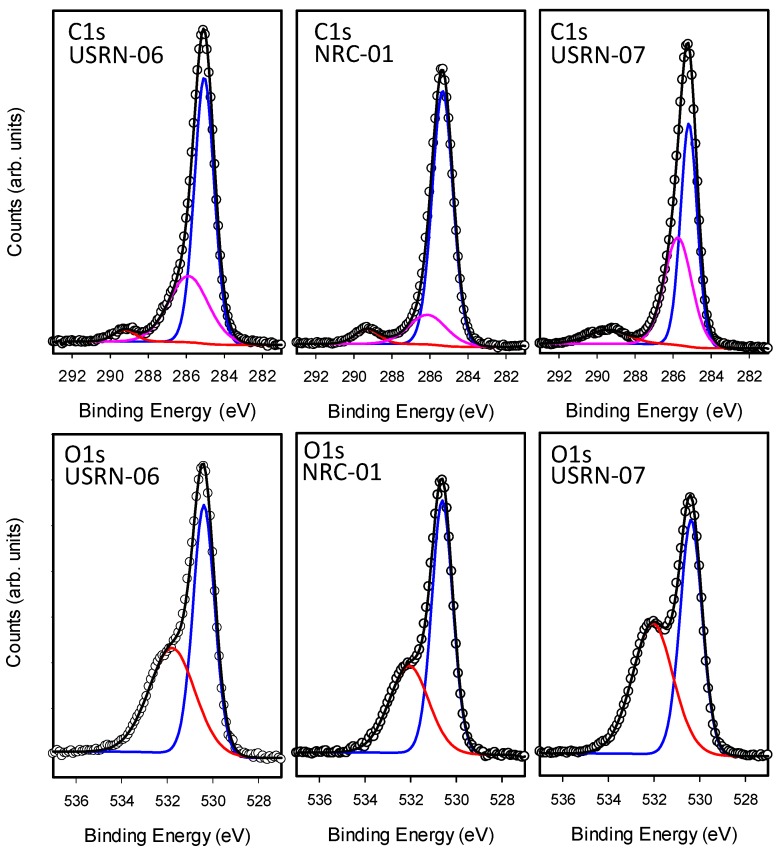
XPS spectra of the C1s (top) and O1s regions for 3 stearic acid-modified ZnO samples. The spectra are fit with a Shirley background and mixed Gaussian-Lorentzian peaks (GL30) using CASA-XPS software. The variously colored peaks correspond to the components in Table 3; C1s blue (C1), pink (C2), red (C3) and dark red (C4), O1s blue (O2) and red (O3).

As for the bare particles, the high-resolution spectra in the C1s and O1s regions were analyzed to identify contributions of different chemical species to the overall carbon and oxygen signals. The spectra and fits are shown in Figure 7 and Figure S7 and the results are summarized in Table 4. In the C1s region, the modified particles all exhibited a large C1 (285 eV) signal which was usually the majority contribution (50–70%). Most of the samples also exhibited C2 and C3 features, although the latter signal was not observed for either of the two caprylsilane-modified samples, even though it was evident in the bare samples from the same source. However, BASF-02-C8 had an additional shoulder at lower binding energy, C0 (283.5 eV), shown in Figure S8. This feature is assigned to Zn-C [34], but was not detected in the other caprylsilane-modified sample (JRC-02). 

There is again no obvious correlation with the TGA results since BASF-02-C8 had the lower overall mass loss and C/Zn ratio, despite the presence of this new XPS signal. The BASF-02-C8 particles also exhibit unusual features in the Zn2p region. The Zn2p signal for ZnO exhibits two peaks separated by 23 eV due to spin-orbit splitting. For most of the particles studied here the Zn region can be fit with two components Zn2p3/2 and Zn2p1/2 with the latter having half the intensity of the former. For BASF-02-C8, this region is best fit with an additional spin-orbit pair shifted to lower binding energy by ~2 eV (see Figure S9). This feature is not observed on JRC-02-C8 or any of the bare particles but has been seen for C doped ZnO produced by calcination [34]. The observation of signals consistent with Zn-C bonds in both the Zn2p and C1s regions provides strong evidence for the formation of carbon-doped ZnO during TGA analysis although it is not clear why such species are only formed for BASF-02-C8. This sample does exhibit a new lower energy signal O1 (529 eV) in the O1s region. Less intense C0 and O1 features are also observed in the first measurement of USRN-05-NH_2_ (but not in the 2^nd^ measurement) suggesting it may be due to extrinsic contamination. Figure 7 shows the spectra for the three stearic acid modified samples. Interestingly, USRN-07-C18, the sample with the large 260 °C TGA mass loss has the largest C3 signal and the largest O3 signal in the C1s and O1s regions, respectively. This further supports our assignment of this species to a carbonate.

### 3.6. In Situ Quantification of Stearic Acid Using ssNMR

**Figure 8:**
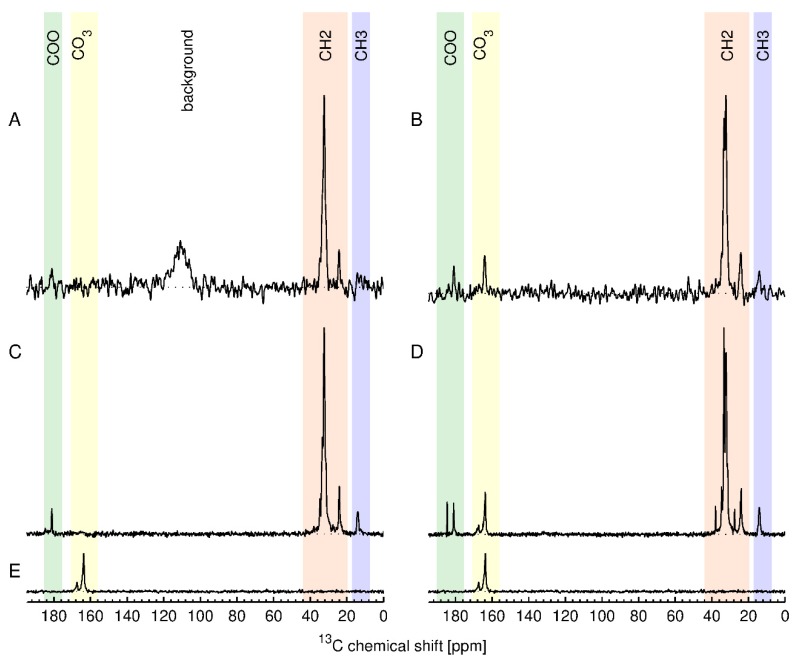
^13^C solid-state NMR spectra of USRN-06-C18 (**A**,**C**), USRN-07-C18 (**B**,**D**) and USRN-02 (**E**). (**A**,**B**) are direct polarization (DP) spectra, whereas (**C**–**E**) are cross-polarization (CP) spectra.

Although ssNMR is a time-consuming method, it is one of the few methods that provides both structural and quantitative information for the intact nanomaterial. Therefore, two of the stearic acid modified samples were also examined by ^13^C ssNMR, along with one unmodified ZnO. We reasoned that the high total carbon content for the stearic acid modified samples should give the maximum sensitivity for direct detection of the functional group. The results are shown in Figure 8 together with their assignment. The top row (A, B) shows ^13^C ssNMR spectra obtained using the inherent direct polarization (DP) of the ^13^C nuclei in the external magnetic field. The obtained spectral signal intensity is directly proportional to the number of ^13^C nuclear spins contributing to the respective signal. This allows to absolutely quantify the different components in the sample by comparing the signal intensity to an external standard [35]. The second and third rows (C, D, E) in Figure 8 show ^13^C cross-polarization (CP) spectra, which are obtained by transferring nuclear polarization from the abundant protons to the ^13^C nuclei, significantly enhancing the sensitivity of the resulting ^13^C spectra. However, these spectra are in general not absolutely quantitative. The spectra of USRN-06-C18 show the expected signals for stearic acid whereas the spectra of USRN-07-C18 have additional signals in the COO and the CH_2_ region. Furthermore, the spectra of both USRN-07-C18 and unmodified ZnO show signals at 164 and 167 ppm that were assigned to carbonate and/or bicarbonate ions on the ZnO surface. The presence of additional ssNMR signals is consistent with the large mass loss at 260 °C for this sample, indicating that the low temperature mass loss is likely to be due to the presence of carbonate. The concentration of the stearic acid groups in USRN-06-C18 and USRN-07-C18 was determined to be 172 µmol/g and 277 µmol/g, respectively, obtained by integration of the CH_2_ signals in the direct polarization experiment. The estimate for USRN-06-C18 is slightly lower than the qNMR value (208 ± 18 µmol/g,), although that for USRN-07-C18 is significantly higher than that determined by qNMR (185 ± 34 µmol/g,). Note that the signal-to-noise ratio for the DP experiment will result in an error on the order of 7–10% (20–30 µmol/g) which may partially account for these differences. 

## 4. Discussion

The present study employed a combination of TGA coupled with analysis of FT-IR spectra of evolved gases and solution ^1^H qNMR to quantify the functional group/coating content in 9 surface modified ZnO NPs; an equivalent number of bare samples from the same sources were examined for comparison. Two additional methods, XPS and ssNMR were used to provide confirmation of the identity of the various components that contribute to the TGA mass loss. Bare NPs that have not been intentionally surface-modified were examined by TGA and had mass losses over the temperature range of 200–900 °C that varied between 0.3 and 5.5%. FT-IR analysis of the evolved gases showed predominantly loss of water and CO_2_, although ammonia (presumably from the synthesis) and signals consistent with the presence of polycylic aromatic hydrocarbons were also detected in one or more samples. A large mass loss at relatively low temperature (inflection point at 260 °C) is consistent with the presence of carbonates (or bicarbonate) based on the detection of XPS and ssNMR signals that can be assigned to these species. Carbonates may be introduced by the synthetic route or by adsorption of atmospheric CO_2_ during storage and handling. It is important not to include this signal with the higher temperature mass loss due to the functional group of interest. Interestingly there is no direct correlation of total mass loss with supplier, as far as can be determined from the samples examined in the present study. 

ZnO nanoparticles that have been surface modified with APTES, caprylsilane and stearic acid were initially examined by qNMR. The qNMR quantification relies on quantitative release of the functional group which required optimization for each of the three surface chemistries. The 3-aminopropylsiloxane was released by basic hydrolysis in water, whereas the use of methanol co-solvent was necessary for quantification of the more hydrophobic caprylsiloxane. Note that methods based on attempted complete dissolution of the ZnO, as in our previous method for silicas NPs, were less successful. Stearic acid was strongly bound to ZnO, possibly covalently, and required use of TFA for complete disintegration of the nanoparticles. This is in contrast to the removal of stearic acid from silica which could be accomplished with solvent extraction under mild conditions. Overall these results indicate that it is challenging to develop an all-purpose method that is compatible with quantitative release of functional groups or surface coatings from ZnO NPs. This is in contrast to our earlier work on silica NPS which demonstrated that the silica dissolution method was compatible with a range of functional group structures with minor adjustments of hydrolysis conditions or addition of a cosolvent to solubilize hydrophobic moieties [19,20]. 

TGA with FT-IR measurement of evolved gases was used to examine each family of modified ZnO NPs. The loss of functional group was confirmed to occur in the 300–600 °C temperature region for all 3 types of materials and there were additional signals at both lower and higher temperature in some cases. The agreement between qNMR and TGA is reasonably good in some cases, particularly for the larger stearic acid and caprylsilanes and for high loading of aminopropylsiloxane. Given the variability in mass loss for bare samples, even from a single supplier, it is challenging to correct for the anticipated mass loss in the same region as the functional group. In some instances (e.g., when the overall functional group loading is low), it is clear that neither the corrected or uncorrected TGA estimate agrees with the qNMR data. This highlights again our previous conclusion [20] that TGA will only provide quantitative estimates of surface functional group content when the loading is high and/or the functional group mass is a significant fraction of the total sample, either due to a high loading or a high molecular weight functional group (eg, a polymer). Note that an assessment of organic content from TGA would be even more challenging for these ZnO samples in the absence of any information as to whether the mass loss occurring below or above the 300–600 °C region should also be assigned to the functional group of interest. Additional contaminants were not identified by solution ^1^H-NMR, although the combination of XPS and ssNMR indicated the presence of carbonate and/or bicarbonate as the source of the low temperature (260 °C) mass loss. XPS has the advantage of providing characteristic signals for carbon and oxygen in different chemical environments, but can also be challenging to interpret due to the prevalence of signals due to adventitious carbon contaminants. 

Generally speaking, the data presented here indicate that the commercial ZnO NPs contain variable levels of carbon and oxygen containing impurities. This makes it challenging to assess surface chemistry by TGA, even with FT-IR of evolved gases. qNMR appears to be a workable solution but does require optimization in order to achieve quantitative recovery of the functional group. Despite the utility of solution ^1^H qNMR for assessing the structure of the functional group, it was necessary to employ additional methods (ssNMR and XPS) to help identify impurities. This highlights the necessity for a multi-method approach for quantification of surface functional groups on commercial nanomaterials. 

Although there are still relatively few studies that quantify the extent of surface modification of nanomaterials, a recent study aimed at identifying organic coatings on 24 different nanomaterials provides a very useful point of comparison [4]. This work employed a combination of TGA and solvent extraction/mass spectrometry (MS) to examine organic coatings on a variety of metal oxides, carbonates and clays sourced from the OECD test program and commercial sources. TGA provided an initial screen to test for the presence of organics and was assumed to provide an estimate of the quantity of surface coating. Solvent extraction and MS were then used to identify the structure. This study met with mixed success, with the TGA extraction, separation and MS approach being successful for 14 of the materials. The remaining 10 materials showed no organic coating by MS, despite mass losses of 2–4% from TGA. The authors summarized the assumptions of their approach as follows: the TGA mass loss after subtraction of water can be equated to loss of the organic coating; the organic coating is fully extractable; the composition of organic in a single extraction is representative of the entire organic content; MS can identify all components; and the particle core is purely inorganic. They also provided a critical assessment of the validity of these assumptions for various materials, an excellent introduction for those wishing to develop methods for quantification of surface chemistry. Our approach was similar except that we have chosen to employ NMR for structural analysis and have worked with a single type of material with multiple functional groups. The present study illustrates the extent of optimization that may be needed for accurate determination of the content of functional groups or coatings and identification of impurities. These results also provide some insight into probable causes for the discrepancies between TGA and MS noted in the previous study [4].

The above study [4] also examined sample JRC-02-C8 and reported a mass loss of 2.1% from TGA; only 10% of this could be accounted for by the trimethoxyalkylsilane detected by MS after solvent extraction [4]. This product was hypothesized to be derived from reaction of the initial silane at the temperature used for solvent extraction. This result compares well to the total mass loss of 1.9% measured here (with 1.3% assigned to the caprylsilane) and values of 2.4% and 1% from previous determinations [25]. Note that the milder extraction conditions used here allowed recovery of the intact caprylsiloxane, providing additional confidence in the method. 

Finally it is useful to compare our data to a recently published study that examined batch-to-batch variability of NP physicochemical properties for silica, cerium dioxide, titanium dioxide and zinc oxide nanoparticles [27]. The materials were all synthesized in-house by the 14 participating laboratories using one or more carefully controlled methods for each material and were characterized for all OECD recommended physicochemical parameters. This study found that some parameters such as zeta potential and size were relatively stable across the materials tested whereas others such as impurities and generation of reactive oxygen species were considerably more variable. This work also provided recommendations on the most appropriate methods for various parameters and suggestions for reducing batch-to-batch variability. Although this study did not include surface modified nanomaterials, the overall conclusion on the prevalence of batch-to batch variability is a common theme, independent of whether materials are produced in house or purchased from a commercial supplier. It will be of considerable interest to compare this study to characterization of a range of commercial nanomaterials, work that is currently underway in our laboratory. 

## 5. Conclusions

A set of commercial zinc oxide nanomaterials, including two from the JRC Nanomaterial Repository, have been characterized by a combination of qNMR in solution and TGA coupled to FT-IR measurements of evolved gases. The materials included surface-modified zinc oxides with 3-aminopropyl, capryl and stearic acids groups, as well as unmodified zinc oxides from the same commercial sources. The qNMR method provides the most reliable method for measuring the total functional group content, although it does require careful optimization of the method for release of the functional group from the surface. TGA correlates reasonably well with NMR data for larger functional groups and high surface loadings. However, TGA does not have adequate sensitivity for analysis of low surface loadings and is complicated by the presence of contaminants, which vary from sample to sample, making it challenging to correct for their presence. One of the contaminants is likely to be carbonate or bicarbonate based on a combination of XPS and ssNMR experiments. The level of surface loading for the various functional groups varies for different suppliers and also for different sizes of material from the same supplier. Overall these results illustrate the importance of developing reliable methods to quantify surface groups. Such methods will be required to ensure reproducible production of materials for a variety of applications and to facilitate nanosafety studies. 

## Figures and Tables

**Table 1:** Thermogravimetric analysis of unmodified ZnO NPs from various suppliers.

Sample (n) ^1^	Size (sd) ^2^, nm	Mass Loss, %		
		200–300 °C	300–600 °C	>600 °C
JRC-01	128 (158) ^3^	0.48	0.19	0.02
BASF-01 (n = 2)	53 (23)	0.03	0.13 ± 0.01	0.11 ± 0.02
USRN-01 (n = 3)	35–45	0.28 ± 0.02	1.60 ± 0.04	1.3 ± 0.3
USRN-02 (n = 2)	18	4.61 ± 0.04	0.88 ± 0.05	0.13 ± 0.01
USRN-03	10–30	0.69	0.48	0.13
NAM-01 (n = 3)	30	0.59 ± 0.03	0.41 ± 0.03	0.08 ± 0.05
NAM-02	20	0.22	0.44	0.21
Sky-01	10–30	0.24	0.22	0.19

^1^ Sample codes are explained in the Materials and Methods. The number of replicate runs (n) are provided in parentheses when multiple samples were analyzed and the standard deviation for the mass loss is provided. ^2^ Nominal particle diameter and standard deviation (sd), as reported by the manufacturer unless otherwise noted. ^3^ Mean diameter measured by TEM from the JRC characterization report [21]; reference [25] reports polyhedral NPs with mean ± sd of 73 ± 32 nm and 38 ± 19 nm for length and height, respectively.

**Table 2:** Quantification of surface coatings on ZnO NPs by qNMR and TGA.^1^

Sample ID	Size, nm	qNMR, μmol/g	TGA Mass Loss, %			TGA, µmol/g ^2^
			200–300 °C	300–600 °C	>600 °C	
USRN-04-NH_2_	20	14.7 ± 0.8 (n = 7)	0.59 (n = 2)	0.90	0.50	154 ± 1 (N/A)
USRN-05-NH_2_	10–30	454 ± 56 (n =3)	4.74 (n = 2)	2.21	4.45	381 ± 51 (299 ± 51)
NAM-03-NH_2_	30	35.2 ± 0.7 (n = 3)	0.25 (n = 2)	0.36	0.03	62 ± 5 (N/A)
JRC-02-C8		102 ± 3 (n = 3)	0.25 (n = 3)	1.31	0.32	110 ± 4 (98 ± 4)
BASF-02-C8	97.5	101 ± 5 (n = 4)	0.1 (n = 2)	1.19	1.1	104 ± 0 (93 ± 0)
USRN-06-C18	18	243 ± 37 (n = 4)	1.32 (n = 2)	4.21	2.62	148 ± 26 (117 ± 26)
USRN-07-C18	10–30	155 ± 44 (n = 4)	5.3 (n = 3)	2.91	3.61	102 ± 34 (86 ± 36)
Sky-02-C18	10–30	34 ± 2 (n = 4)	4.74 (n = 2)	1.61	0.75	57 ± 18 (49 ± 18)
NRC-01-C18 ^3^		93 ± 6 (n=4)	0.19 (n = 3)	1.88	1.98	65 ± 2 (61 ± 1)

^1^ The number of replicate samples, n, is indicated in parentheses for qNMR and TGA quantifications. The details of the extraction method used for each sample are summarized in the Materials and Methods section and the Supplementary Material. ^2^ The functional group content based on the mass loss between 300–600 °C is the 1st entry for each sample. The corrected value obtained by subtracting the mass loss for bare NPs of the same size is the 2^nd^ entry. Cases in which the correction is clearly in error (i.e., gives a negative number) are noted as NA. ^3^ Prepared from BASF-01.

**Table 3:** Relative atomic compositions obtained from quantitative analysis of XPS data ^1^ for unmodified ZnO NPs.

	O/Zn	C/Zn	C1	C2	C3	C4	O2	O3
			% of Total Carbon Signal ^2^				% of O Signal ^3^	
**JRC-01**	**1.05**	**0.42**	**67.2**	**21.5**	**6.1**	**5.3**	**59.0**	**41.0**
	0.02	0.02	2.9	2.2	0.8	1.6	1.1	1.1
**BASF-01**	**1.03**	**0.67**	**77.9**	**17.3**	**4.8**		**58.9**	**41.1**
	0.04	0.05	1.4	2.5	1.1		0.9	0.9
	**1.05**	**0.44**	**68.5**	**19.0**	**8.3**	**4.2**	**61.5**	**38.5**
	0.05	0.05	1.9	0.6	0.8	2.7	**2.2**	**2.2**
**USRN-01**	**0.99**	**0.40**	**71.0**	**21.2**	**7.8**		**58.0**	**42.0**
	0.00	0.01	9.7	8.6	1.1		0.4	0.4
**USRN-02**	**1.18**	**0.99**	**58.8**	**32.7**	**8.5**		**52.4**	**47.6**
	0.01	0.04	3.6	3.5	0.1		0.8	0.8
**NAM-01**	**1.06**	**0.39**	**71.3**	**11.1**	**7.1**	**10.5**	**58.4**	**41.6**
	0.04	0.03	1.6	1.8	0.7	0.7	1.1	1.0

^1^ For each sample, the composition is shown in bold on the first row, with the standard deviation based on measurements from three separate areas provided below. ^2^ Carbon signals are peaked at 285 eV, 286 eV, 289 eV and 290 eV for C1, C2, C3 and C4, respectively. ^3^ Oxygen signals are peaked at 530.5 eV and 532 eV for O2 and O3 respectively.

**Table 4:** Relative atomic compositions obtained from quantitative analysis of XPS data ^1^ for surface-modified ZnO NPs.

	O/Zn	C/Zn	Si2s/Zn	Si2p/Zn	N/Zn	C0	C1	C2	C3	O1	O2	O3
Stearic Acid						% of Carbon Signal ^2^				% of Oxygen Signal ^3^		
USRN-06-C18	**1.35**	**2.57**					**73.4**	**22.1**	**3.3**		**54.8**	**45.3**
	0.09	0.31					1.3	1.4	0.3		2.6	2.6
NRC-01-C18	**1.19**	**1.53**					**78.1**	**17.2**	**4.8**		**61.0**	**39.0**
	0.04	0.12					3.4	3.4	0.5		1.1	1.1
USRN-07-C18	**1.45**	**2.03**					**51.7**	**40.5**	**7.9**		**48.4**	**51.6**
	0.17	0.43					6.5	5.5	1.0		2.2	2.2
Capryl silane												
BASF-02-C8	**1.12**	**0.92**	**0.092**	**0.115**		**34.1**	**63.7**	**2.2**		**26.7**	**51.4**	**21.9**
	0.04	0.01	0.014	0.028		0.5	0.6	0.1		1.9	1.2	3.2
JRC-02-C8	**1.37**	**0.99**	**0.083**	**0.094**			**70.8**	**29.2**			**49.3**	**50.7**
	0.03	0.02	0.004	0.006			9.5	9.5			1.0	1.0
APTES												
USRN-05-NH_2_	**1.26**	**0.87**	**0.122**	**0.123**	**0.081**	**11.3**	**59.9**	**24.9**	**3.9**	**7.1**	**55.3**	**37.7**
	0.05	0.04	0.007	0.001	0.009	15.5	9.6	5.9	0.1	6.6	3.1	3.6
	**1.37**	**0.56**	**0.077**	**0.092**	**0.088**		**41.0**	**50.8**	**8.2**		**43.1**	**56.9**
	0.02	0.00	0.009	0.005	0.006		3.3	3.0	0.7		6.6	6.6

^1^ For each sample, the composition is shown in bold on the first row, with the standard deviation based on measurements from three separate areas provided below. ^2^ Carbon signals are peaked at 283 eV, 285 eV, 286 eV and 289 eV for C0, C1, C2 and C3, respectively. ^3^ Oxygen signals are peaked at 529 eV, 530.5 eV and 532 eV for O1, O2 and O3, respectively.

## Supplementary Materials

The following are available online at https://www.mdpi.com/2079-4991/10/4/678/s1: Figures S1–S9 with additional thermograms, FT-IR spectra and XPS spectra. Tables S1–S12 with details on the optimization of functional group or coating release from ZnO NPs and XPS data from survey scans.

## References

[1] Heuer-Jungemann A., Feliu N., Bakaimi I., Hamaly M., Alkilany A., Chakraborty I., Masood A., Casula M.F., Kostopoulou A., Oh E. (2019). The role of ligands in the chemical synthesis and applications of inorganic nanoparticles. Chem. Rev..

[2] Grassian V.H. (2008). When size really matters: Size-dependent properties and surface chemistry of metal and metal oxide nanoparticles in gas and liquid phase environments. J. Phys. Chem. C.

[3] Krug H.F. (2014). Nanosafety research - Are we on the right track?. Angew. Chem. Int. Ed..

[4] Clausen P.A., Kofoed-Sørensen V., Nørgaard A.W., Sahlgren N.M., Jensen K.A. (2019). Thermogravimetry and mass spectrometry of extractable organics from manufactured nanomaterials for identification of potential coating components. Materials.

[5] Baer D.R. (2018). The chameleon effect: Characterization challenges due to the variability of nanoparticles and their surfaces. Front. Chem..

[6] Baer D.R., Gaspar D.J., Nachimuthu P., Techane S.D., Castner D.G. (2010). Application of surface chemical analysis tools for characterization of nanoparticles. Anal. Bioanal. Chem..

[7] Hennig A., Borcherding H., Jaeger C., Hatami S., Wurth C., Hoffmann A., Hoffmann K., Thiele T., Schedler U., Resch-Genger U. (2012). Scope and limitations of surface functional group quantification methods: Exploratory study with poly(acrylic acid)-grafted micro and nanoparticles. J. Am. Chem. Soc..

[8] Hennig A., Dietrich P.M., Hemmann F., Thiele T., Borcherding H., Hoffmann A., Schedler U., Jäger C., Resch-Genger U., Ungera W.E.S. (2015). En route to traceable reference standards for surface group quantifications by XPS, NMR and fluorescence spectroscopy. Analyst.

[9] Moser M., Nirmalananthan N., Behnke T., Geibler D., Resch-Genger U. (2018). Multimodal cleavable reporters versus conventional labels for optical quantification of accessible amino and carboxy groups on nano- and microparticles. Anal. Chem..

[10] Ambrogio M.W., Frasconi M., Yilmaz M.D., Chen X. (2013). New methods for improved characterization of silica nanoparticle-based drug delivery systems. Langmuir.

[11] Crucho C.I.C., Baleizao C., Farinha J.P.S. (2017). Functional group coverage and conversion quantification in nanostructured silica by ^1^H NMR. Anal. Chem..

[12] Das D., Yang Y., O’Brien J.S., Breznan D., Nimesh S., Bernatchez S., Hill M., Sayari A., Vincent R., Kumararathasan P. (2014). Synthesis and physicochemical characterization of mesoporous SiO_2_ nanoparticles. J. Nanomat..

[13] Hsiao I.-L., Fritsch-Decker S., Leidner A., Al-Rawi M., Hug V., Diabate S., Grage S.L., Meffert M., Stoeger T., Gerthsen D. (2019). Biocompatibility of amine-functionalized silica nanoparticles: The role of surface charge. Small.

[14] Kong N., Zhou J., Park J., Xie S., Ramstrom O., Yan M. (2015). Quantitative fluorine NMR to determine carbohydrate density on glyconanomaterials synthesized from perfluorophenyl azide-functionalized silica nanoparticles by click reaction. Anal. Chem..

[15] Lehman S.E., Tataurova Y., Mueller P.S., Mariappan S.V., Larsen S.C. (2014). Ligand characterization of covalently functionalized mesoporous silica nanoparticles: An NMR toolbox approach. J. Phys. Chem. C.

[16] Soto-Cantu E., Cueto R., Koch J., Russo P.S. (2012). Synthesis and rapid characterization of amine-functionalized silica. Langmuir.

[17] Sun Y., Kunc F., Balhara V., Coleman B., Kodra O., Reza M., Chen M., Brinkmann A., Lopinski G.P., Johnston L.J. (2019). Quantification of amine functional groups on silica nanoparticles: A multi-method approach. Nanoscale Adv..

[18] Hristov D.R., Rocks L., Kelly P.M., Thomas S.S., Pitek A.S., Verderio P., Mahon E., Dawson K.A. (2015). Tuning of nanoparticle biological functionality through controlled surface chemistry and characterisation at the bioconjugated nanoparticle surface. Sci. Rep..

[19] Kunc F., Balhara V., Brinkmann A., Sun Y., Leek D.M., Johnston L.J. (2018). Quantification and stability of surface amine groups on silica nanoparticles using solution NMR. Anal. Chem..

[20] Kunc F., Balhara V., Sun Y., Daroszewska M., Jakubek Z.J., Hill M., Brinkmann A., Johnston L.J. (2019). Quantification of surface functional groups on silica nanoparticles: Comparison of thermogravimetric analysis and quantitative NMR. Analyst.

[21] Singh C., Friedrichs S., Levin M., Birkedal R., Jensen K.A., Pojana G., Wohlleben W., Schulte S., Wiench K., Turney T. (2011). NM-Series of Representative Manufactured Nanomaterials: Zinc Oxide NM-110, NM-111, NM-112, NM-113. Characterisation and Test Item Preparation.

[22] Farcal L., Torres Andon F., Di Cristo L., Rotoli B.M., Bussolati O., Bergamaschi E., Mech A., Hartmann N.B., Rasmussen K., Reigo-Sintes J. (2015). Comprehensive in vitro toxicity testing of a panel of representative oxide nanomaterials: First steps towards an intelligent testing strategy. PLoS ONE.

[23] Kołodziejczak-Radzimska A., Jesionowski T. (2014). Zinc Oxide—From Synthesis to Application: A Review. Materials.

[24] Fung B.M., Khitrin A.K., Ermolaev K. (2000). An improved broadband decoupling sequence for liquid crystals and solids. J. Magn. Reson..

[25] Yin H., Coleman V.A., Casey P.S., Angel B., Catchpole H.J., Waddington L., McCall M.J. (2015). A comparative study of the physical and chemical properties of nano-sized ZnO particles from multiple batches of three commercial products. J. Nanopart. Res..

[26] Wang N., Tong T., Xie M., Gaillard J.-F. (2016). Lifetime and dissolution kinetics of zinc oxide nanoparticles in aqueous media. Nanotechnology.

[27] Mülhopt S., Diabaté S., Dilger M., Adelhelm C., Anderlohr C., Bergfeldt T., Torre J.G.D.I., Jiang Y., Valsami-Jones E., Langevin D. (2018). Characterization of nanoparticle batch-to-batch variability. Nanomaterials.

[28] Gabka G., Bujak P., Giedyk K., Kotwica K., Ostrowski A., Malinowska K., Lisowski W., Sobczakc J.W., Prona A. (2014). Ligand exchange in quaternary alloyed nanocrystals–a spectroscopic study. Phys. Chem. Chem. Phys..

[29] Brassard J.-D., Sarkar D.K., Perron J. (2015). Studies of drag on the nanocomposite superhydrophobic surfaces. Appl. Surf. Sci..

[30] Winiarski J., Tylus W., Winiarski K., Szczygiel I., Szcygiel B. (2018). XPS and FT-IR Characterization of Selected Synthetic Corrosion Products of Zinc Expected in Neutral Environment Containing Chloride Ions. J. Spect..

[31] Shchukarev A.V., Korolkov D.V. (2004). XPS Study of Group IA Carbonates. Central Eur. J. Chem..

[32] Wallace R., Brown A.P., Brydson R., Wegner K., Milne S.J. (2013). Synthesis of ZnO nanoparticles by flame spray pyrolysis and characterization protocol. J. Mater. Sci..

[33] Wang L., Muhammed M. (1999). Synthesis of zinc oxide nanoparticles with controlled morphology. J. Mater. Chem..

[34] Pan L., Muhammad T., Ma L., Huang Z.-F., Wang S., Wang L., Zou J.-J., Zhang X. (2016). MOF-derived C-doped ZnO prepared via a two-step calcination for efficient photocatalysis. Appl. Catal. B.

[35] Holzgrabe U., Wawer I., Diehl B. (2008). NMR Spectroscopy in Pharmaceutical Analysis.

