# Supplementary material for: A Multi-Method Approach for Quantification of Surface Coatings on Commercial Zinc Oxide Nanomaterials
Source: Nanomaterials (Basel). 2020 Apr 3;10(4):678. doi: 10.3390/nano10040678 (PMC7221730; doi:10.3390/nano10040678)
Supplement: Supplementary file 1 [file nanomaterials-10-00678-s001.pdf]

Supplementary Material

A multi-method approach for quantification of surface coatings on commercial zinc oxide nanomaterials

Filip Kunc, Oltion Kodra, Andreas Brinkmann, Gregory Lopinski, Linda J. Johnston

National Research Council Canada, Ottawa, ON Canada K1A 0R6

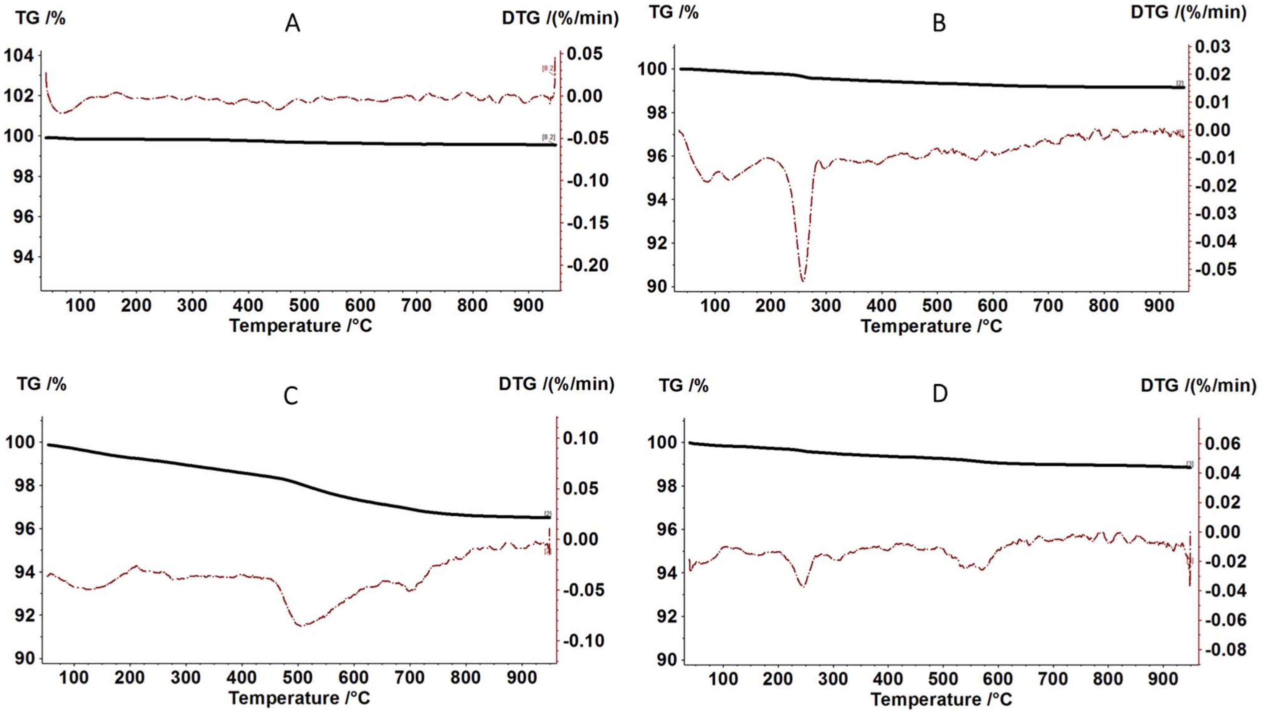

Figure S1: Representative thermograms of bare ZnO samples: A) BASF-01, B) Sky-01, C) USRN-01 and D) NAM-02

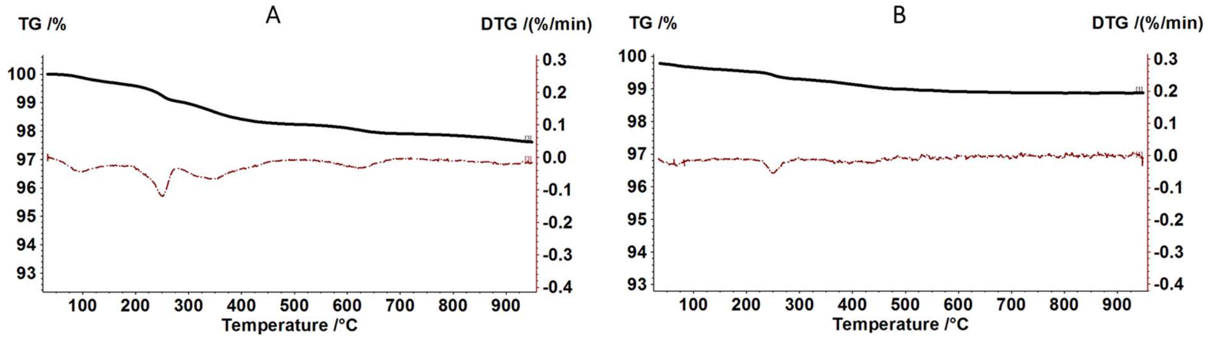

Figure S2: Thermograms for USRN-04-NH_2 (A) and NAM-03-NH_2 (B).

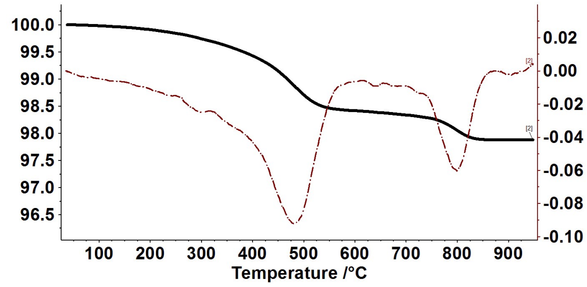

Figure S3: Thermogram for JRC-02-C8

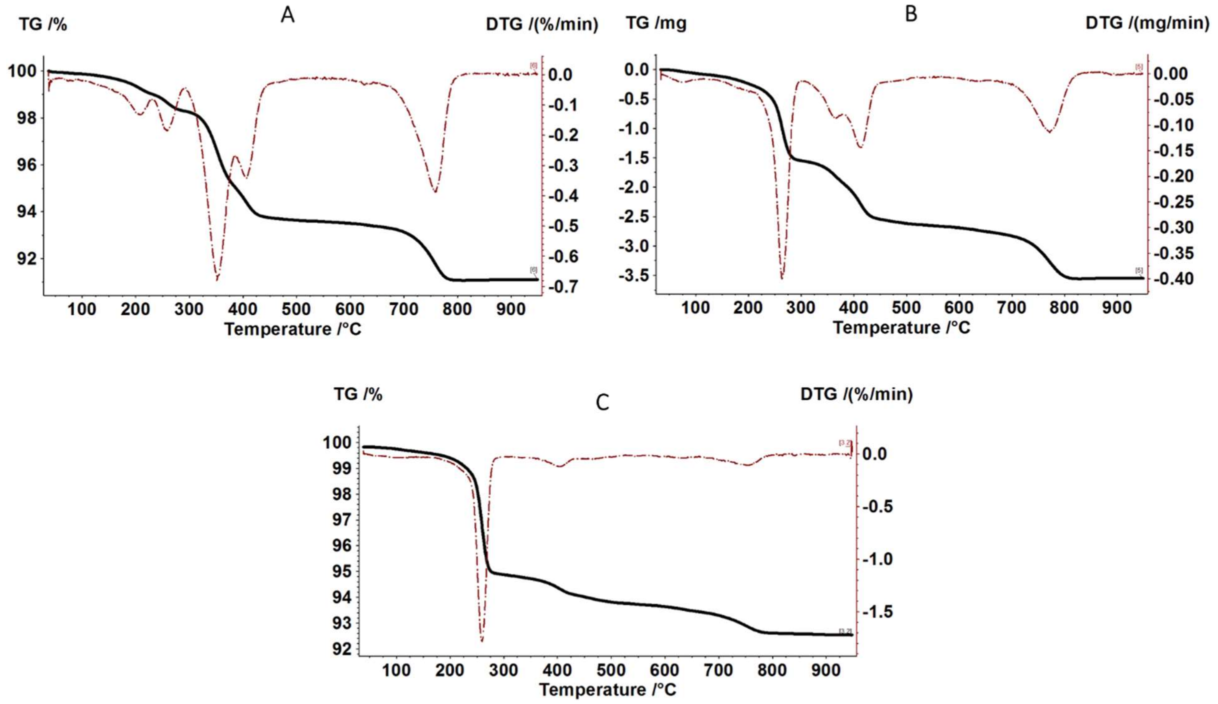

Figure S4: Thermograms of stearic acid modified samples A) USRN-06-C18, B) USRN-07-C18, and C) Sky-02-C18

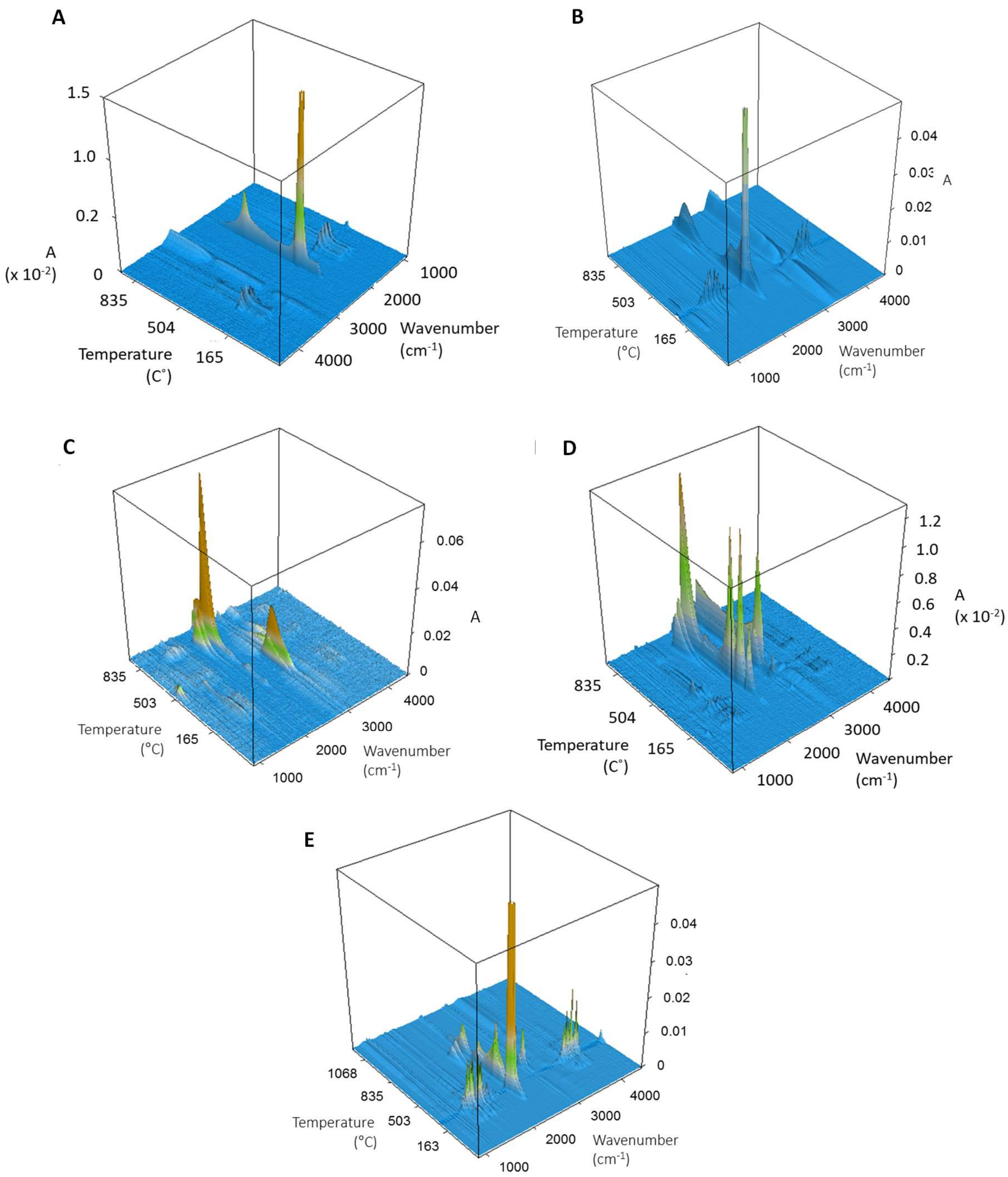

Figure S5: 3D FT-IR spectra of evolved gas during TGA: 3D FT-IR spectra of evolved gas during TGA: A) NAM-01, B) USRN-05-NH_2, C) BASF-02-C8, D) NRC-01-C18, and E) USRN-07-C18

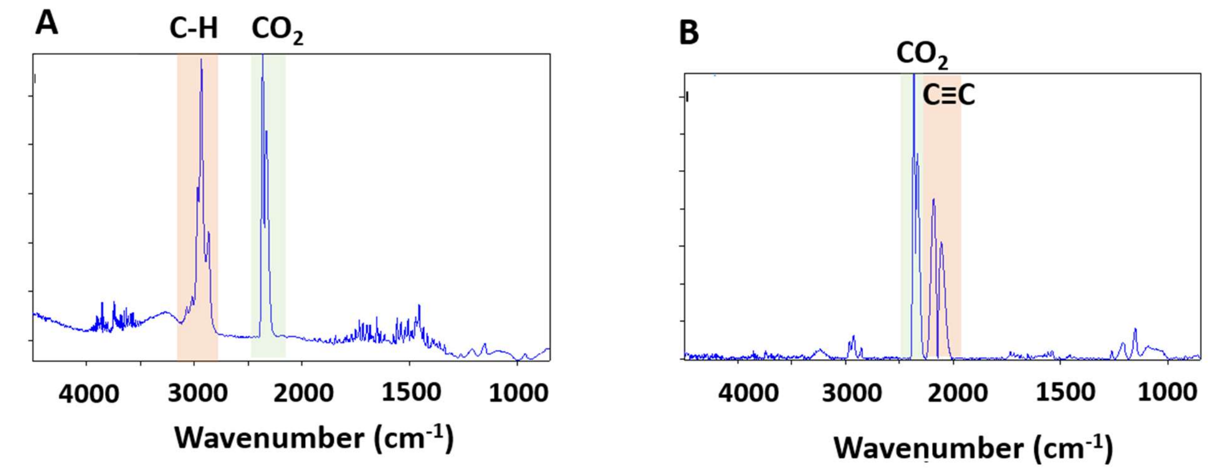

Figure S6: FT-IR spectra of USRN-07-C18 taken at 403 ˚C (A) and 780 ˚C (B)

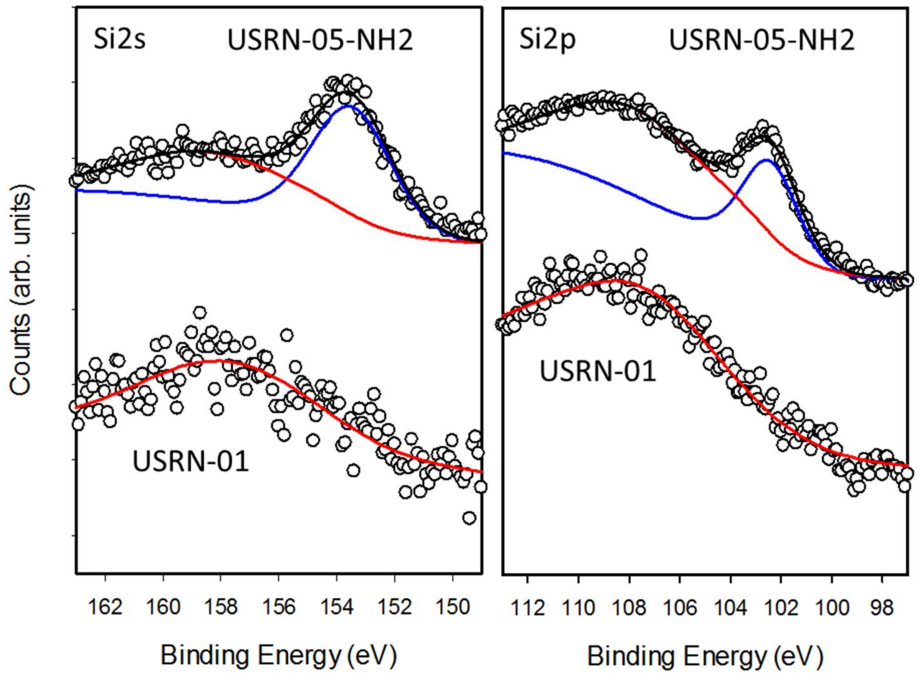

Figure S7: XPS high resolution of Si2s and Si2p regions showing detection of silicon on APTES modified ZnO particles (USRN-05-NH_2). Spectra for bare ZnO particles (USRN-01) are shown as a control. Fits show the decomposition of the spectra into two features, the sharp Si2s and Si2p components in blue and the broad plasmon loss feature associated with the Zn3s and Zn3p peaks (not shown).

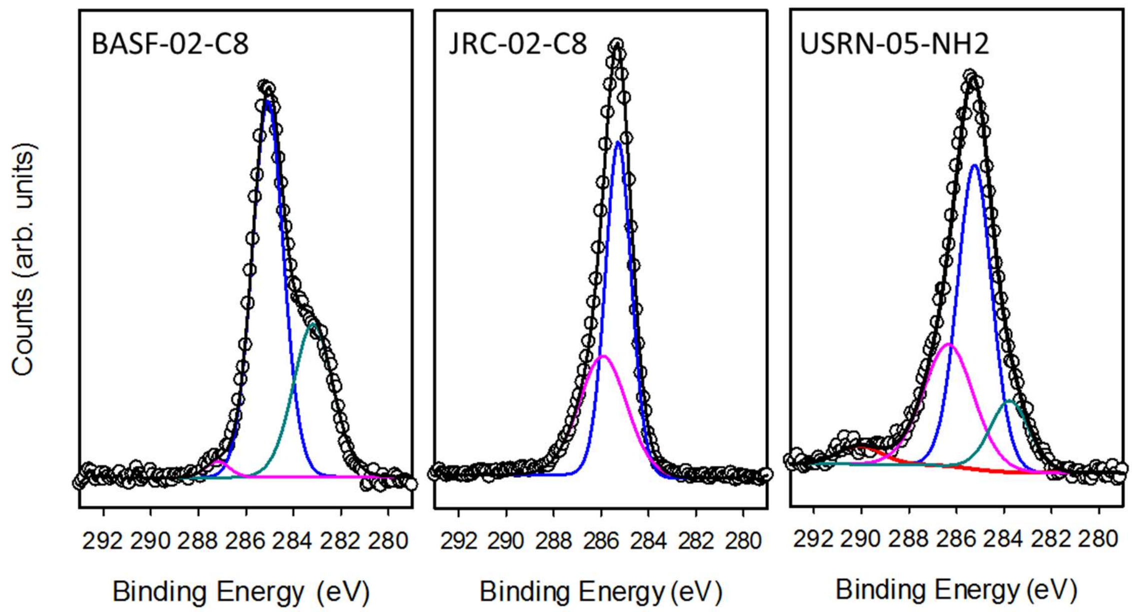

Figure S8: XPS high resolution spectra of C1s region for two caprylsilane and one APTES modified ZnO samples. Curve fits to decompose the spectra into contributions from CO (cyan), C1 (blue), C2 (pink) and C3 (red) are also shown.

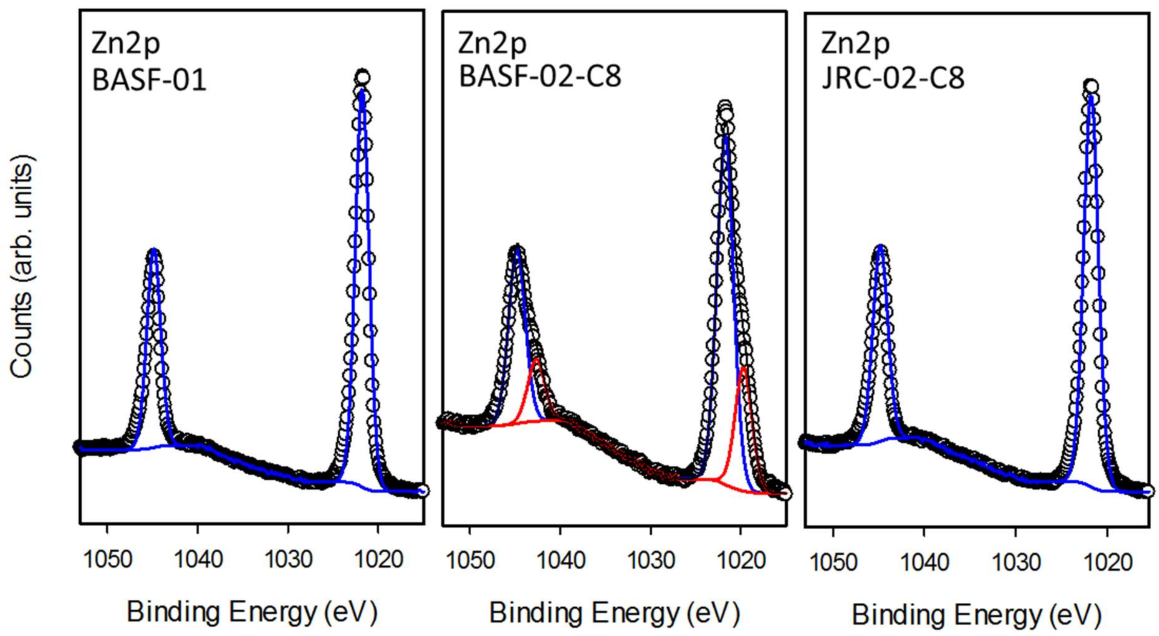

Figure S9: XPS high resolution spectra of Zn2p region for a bare ZnO sample (BASF-01) and the two caprylsilane modified samples. The blue curves represent fits to the usual 2p3/2 and 2p1/2 features while the red curves represent an additional downshifted spin-orbit doublet observed on the BASF-02-C8 sample.

Tables. Note that the underlined average values at the end of Tables S1-S10 indicate the functional group content based on averaging all experiments using the optimized procedure. Standard deviations for individual experiments are based on averaging the NMR signals used for quantification.

Table S1: Summary of sample preparation conditions for the qNMR quantification of sample NAM-03-NH_2. The standard deviation for the individual runs is calculated from the three NMR integral values. In some cases a second extraction was carried out. Additional 3-aminopropyl amine is detected in the second NaOD extraction when a large mass of sample is analyzed, but not for sample masses below ~ 10 mg (below limit of detection, BLD). In the case of acidic treatment with 0.4 M DCl, the sample did not fully dissolve unlike other ZnOs. The sample was analyzed as a cloudy suspension and gave a lower amine content than the NaOD extractions.

	**0.4 M DCl**		**0.4 M NaOD**			
	**1st extraction 3 h, 45 ℃ (+2nd extraction 24h)***		**1st extraction 24 h, 45 ℃ (+2nd extraction 24h)**		**1st extraction 78 h, 45 ℃**	
	**Analysed mass (mg)**	**Quantification (µmol/g)**	**Analysed mass (mg)**	**Quantification (µmol/g)**	**Analysed mass (mg)**	**Quantification (µmol/g)**
**1**	13.44	**28.8**<br>**3.9 ***	31.40	**34.9 ± 0.5**<br>**2.0 ± 0.2**	13.13	**34.0**<br>**BLD**
**2**	8.65	**25.1 ±3.7**	19.00	**34.5 ± 0.2**<br>**1.5 ± 0.2**		
**3**	8.37	**21.3 ± 1.1**	6.09	**36.3 ± 1.1**		
**4**	10.87	**20.8 ±0.6**	7.92	**34.8 ± 1.3**<br>**BLD**		
**5**	20.33	**30.2 ± 0.5**	10.57	**34.4 ± 0.3**		
**Avg**		**25.2 ± 3.5**		**<u>35.2 ± 0.7</u>**		**34**

*The undissolved ZnO solids were collected by ultracentrifugation and suspended in another aliquot of DCl 0.4M.

Table S2: Summary of sample preparation conditions for the qNMR quantification of USRN-04-NH_2. Note that due to the relatively low amine content, the second NaOD extraction does not yield a detectable amount of 3-aminopropylsilicate (BLD).

	0.4 M DCl		0.4 M NaOD			
	1st extraction 24 h, 45 ℃		1st extraction 24 h, 45 ℃ (+2nd extraction 24h)		1st extraction 96 h, 45 ℃	
	Analysed mass (mg)	Quantification (µmol/g)	Analysed mass (mg)	Quantification (µmol/g)	Analysed mass (mg)	Quantification (µmol/g)
**1**	11.90	**16.3 ± 1.2**	10.10	**15.4 ± 1.4**	8.41	**15.7 ± 0.9**
**2**	7.95	**17.5 ± 1.6**	7.31	**16.5 ± 1.4**		
**3**			17.71	**15.0 ± 0.8**		
**4**			24.35	**14.2 ± 1.0**		
**5**			36.96	**14.0 ± 1.1**		
**6**			13.75	**14.4 ± 0.9**		
**7**			10.94	**13.7 ± 0.1**<br>**BLD**		
**Avg**		**15.4 ± 1.7**		**14.7 ± 0.8**		**15.7**

Table S3: Summary of sample preparation conditions for the qNMR quantification of USRN-05-NH_2. Due to higher amine loading, the second extraction gave a significant amount of 3-aminopropylsiloxane; however, a third NaOD extraction did not lead to a detectable amount (BLD).

	0.4 M DCl		0.4 M NaOD			
	1st extraction 24 h, 45 ℃		1st extraction 24 h, 45 ℃ (+2nd extraction 24h) (+3rd extraction 24h)		1st extraction 78 h, 45 ℃	
	Analysed mass (mg)	Quantification (µmol/g)	Analysed mass (mg)	Quantification (µmol/g)	Analysed mass (mg)	Quantification (µmol/g)
**1**	9.44	**389.8 ± 3.0**	10.38	**395 ± 1.5**<br>**13.2 ± 0.3**	7.64	**511.3 ± 1.1**
**2**	7.53	**486.7 ± 6.6**	8.11	**380.8 ± 1.5**<br>**24.8 ± 1.1**	7.77	**373.0 ± 2.2**
**3**	6.81	**490.1 ± 5.3**	10.64	**523.0 ± 2.1**<br>**18.9 ± 0.9**<br>**BLD**		

**4**	8.90	**446.7 ± 3.5**				
**Avg**		**453 ± 36**		**432 ± 56**<br>**22 ± 2**		**442 ± 56**
**total**				<u>**454 ± 56**</u>		

Table S4: Summary of sample preparation conditions for the qNMR quantification of BASF-02-C8.

	**0.2 M NaOD**		**0.4 M NaOD**				**0.8 M NaOD**	
	**1st extraction 24 h, 45 ℃**<br>**(+2nd extraction 24h)**		**1st extraction 48 h, 45 ℃**<br>**(+2nd extraction 24h)**<br>**(+3rd extraction 24h)**		**1st extraction 78 h, 45 ℃**		**1st extraction 78 h, 45 ℃**	
	**Analysed mass (mg)**	**Quantification (µmol/g)**	**Analysed mass (mg)**	**Quantification (µmol/g)**	**Analysed mass (mg)**	**Quantification (µmol/g)**	**Analysed mass (mg)**	**Quantification (µmol/g)**
**1**	9.40	**46.1 ± 6.7**<br>**15.2 ±**<br>**5.6 ± 3.4**	12.20	**79 ± 9**	8.03	**84.8 ± 5.5**	11.34 mg	**98.2 ± 2.5**
**2**	11.10	**44.6 ± 2.5**<br>**9.4± 0.2**	6.75	**85.4 ± 4.5**<br>**20.3 ± 4.7**				
**3**			6.82	**90.3 ± 14.9**<br>**17**<br>**trace***				
**4**			7.76	**78.4 ± 12.0**<br>**15.7**<br>**trace***				
**Avg**		**45.3 ± 0.6**<br>**12.3 ± 2.4**		**83.3 ± 4.4**<br>**17.7 ± 1.6**		**84.8**		**98.2**
**total**				<u>**101.0 ± 4.7**</u>				

*Trace amounts were below the limit of quantification

Table S5: Summary of sample preparation conditions for the qNMR quantification of sample JRC-02-C8.

#	0.2 NaOD		0.4 M NaOD		0.8 M NaOD			
	1st extraction 24 h, 45 C (+2nd extraction 24h)		1st extraction 48 h, 45 C (+2nd extraction 24h)		1st extraction 24 h, 45 C		1st extraction 78 h, 45 C	
	Analysed mass (mg)	Quantification (µmol/g)	Analysed mass (mg)	Quantification (µmol/g)	Analysed mass (mg)	Quantification (µmol/g)	Analysed mass (mg)	Quantification (µmol/g)
**1**	12.65<br>12.65	**40.4 ± 3.9**<br>**15.2 ± 2.1**	8.35	**90.1 ± 0.4**	**6.62 mg**	**96.8 ± 12.0**	**7.34**	**96.2 ± 5.4**<br>**13.5 ± 2.2**
**2**	11.10	**39.2 ± 3.3**<br>**13.2 ± 2.7**	7.70	**81.1 ± 2.3**<br>**16.4 ± 1.1**				
**3**			8.21	**87.3 ± 2.9**<br>**16.5 ± 1.4**				
**Avg**		**39.8 ± 0.5**<br>**14.2 ± 0.8**		**86.1 ± 3.3**<br>**16.5 ± 0.1**		**96.8**		**96.2**<br>**13.5***
**total**				**101.6 ± 3.3**				

*due to low S/N the quantification here is less reliable than the quantification of the 0.4 M NaOD second supernatant

Table S6: Summary of various solvent extractions for stearic acid modified ZnO samples.

Sample	Solvent Used	Analysed mass (mg)	1st extract Quantification (µmol/g)	2nd extract Quantification (µmol/g)	3rd extract Quantification (µmol/g)
**Sky-02-C18**	DMSO-d6	8.60	**10.3**		
**USRN-06-C18**	DMSO-d6	14.03	**6.3**	**12**	**11.6**
**USRN-07-C18**	DMSO-d6	10.30	**2.5**	**2.4**	
**NRC-01-C18**	DMSO-d6	9.74	**3.7**	**4.9**	
**NRC-01-C18**	CD_3OD – Pyridine-d4 16%	12.19	**43.4 ± 2.4**	**6.5 ± 0.6**	
**USRN-SiO_2-C18**	DMSO-d6	10.48	**173.2 ± 8.3**	**15.4 ± 1.4**	
**USRN-SiO_2-C18**	CD_3OD	8.38	**133.8 ± 10.4**	**11.3 ± 0.4**	

Table S7: Summary of sample preparation conditions for the qNMR quantification of USRN-06-C18

	**8% TFA in CD_3OD**		**16% TFA in CD_3OD**	
	**Analysed mass (mg)**	**Quantification (μmol/g)**	**Analysed mass (mg)**	**Quantification (μmol/g)**
**1**	9.77	**260.2 ± 10.3**	8.45	**187.0 ± 8.4**
**2**	7.24	**299.0 ± 11.5**	11.59	**229.8 ± 11.0**
**3**	9.95	**237 ± 33.5**		
**Avg**	**242.6 ± 36.8 μmol/g**			

Table S8: Summary of sample preparation conditions for the qNMR quantification of USRN-07-C18

	**8% TFA in CD_3OD**		**16% TFA in CD_3OD**	
	**Analysed mass (mg)**	**Quantification (μmol/g)**	**Analysed mass (mg)**	**Quantification (μmol/g)**
**1**	6.86	**50.6 ± 4.6***	9.86	**142.8 ± 6.0**
**2**	6.70	**109.2 ± 9.0**	10.19	**226.7 ± 10.6**
**3**	7.58	**139.3 ± 10.7**		
**Avg**	**154.5 ± 43.7 μmol/g**			

*excluded as an outlier

Table S9: Summary of sample preparation conditions for the qNMR quantification of Sky-02-C18

	**8% TFA in CD_3OD**		**16% TFA in CD_3OD**	
	**Analysed mass (mg)**	**Quantification (μmol/g)**	**Analysed mass (mg)**	**Quantification (μmol/g)**
**1**	9.02	**36.0 ± 2.6**	11.86	**32.7 ± 2.7**
**2**	10.45	**36.7 ± 3.3**	10.12	**31.6 ± 1.9**
**Avg**	**34.3 ± 2.2 μmol/g**			

Table S10: Summary of sample preparation conditions for the qNMR quantification of NRC-01-C18

	1.5 % TFA in CD_3OD		8% TFA in CD_3OD		16% TFA in CD_3OD	
	Analysed mass (mg)	Quantification (µmol/g)	Analysed mass (mg)	Quantification (µmol/g)	Analysed mass (mg)	Quantification (µmol/g)
**1**	9.76 *	**91.1 ± 3.1**	10.05	**101.3 ± 2.1**	14.22	**85.9 ± 1.1**
**2**					12.46 *	**92.8 ± 2.4**
**Avg**	**92.8 ± 5.5 µmol/g**					

*contains 16% pyridine-d_5

Table S11: % Atomic Composition from XPS survey scans of unmodified ZnO nanoparticles.*

	Zn 2p	O 1s	C 1s	Cl 2p
**USRN-01**	48.8	37.2	10.9	3.1
	0.4	0.8	1.0	0.2
**NAM-01**	46.6	38.1	14.5	0.8
	0.9	0.2	0.6	0.1
**BASF-01 #1**	45.5	36.9	15.9	1.6
	0.4	0.2	0.1	0.2
**#2**	45.3	38.0	15.9	0.8
	0.9	0.1	0.7	0.1
**USRN-02**	42.4	37.2	18.5	1.9
	0.2	1.0	0.7	0.0
**JRC-01**	45.6	37.9	15.6	1.0
	0.2	0.3	0.3	0.1

*The red numbers correspond to the average of measurements from three different spots, and the black numbers are the standard deviation of those measurements.

Table S12: % Atomic Composition from XPS survey scans of modified ZnO nanoparticles.*

	Zn 2p	O 1s	C 1s	Cl 2p	N 1s	Si 2s
**USRN-06-C18**	28.7	30.1	39.9	1.3		
	2.1	0.4	2.0	0.3		
**NRC-01-C18**	28.6	29.3	41.8	0.3		
	0.5	0.1	0.5	0.1		
**USRN-07-C18**	30.8	41.7	27.5			
	2.6	1.9	4.4			
**BASF-02-C8**	33.3	33.7	30.3			2.7
	0.8	0.5	1.4			0.1
**JRC-02-C8**	32.3	35.4	30.3			2.0
	0.4	0.2	0.3			0.3
**USRN-05-NH2 #1**	33.8	37.4	21.2	2.2	3.2	2.3
	0.1	0.3	0.3	0.3	0.2	0.0
**#2**	35.9	39.8	18.2	1.0	3.3	1.9
	0.3	0.7	0.5	0.1	0.1	0.7

*The red numbers correspond to the average of measurements from three different spots, and the black numbers are the standard deviation of those measurements.
